# The Inner Nuclear Membrane Is a Metabolically Active Territory that Generates Nuclear Lipid Droplets

**DOI:** 10.1016/j.cell.2018.05.047

**Published:** 2018-07-26

**Authors:** Anete Romanauska, Alwin Köhler

**Affiliations:** 1 Max F. Perutz Laboratories, Medical University of Vienna, Vienna Biocenter Campus (VBC), Dr. Bohr-Gasse 9/3, 1030 Vienna, Austria

**Keywords:** inner nuclear membrane, endoplasmic reticulum, nuclear lipid droplets, lipid metabolism, lipid sensors, diacylglycerol, phosphatidic acid, Seipin, Lipin, transcription factor

## Abstract

The inner nuclear membrane (INM) encases the genome and is fused with the outer nuclear membrane (ONM) to form the nuclear envelope. The ONM is contiguous with the endoplasmic reticulum (ER), the main site of phospholipid synthesis. In contrast to the ER and ONM, evidence for a metabolic activity of the INM has been lacking. Here, we show that the INM is an adaptable membrane territory capable of lipid metabolism. *S. cerevisiae* cells target enzymes to the INM that can promote lipid storage. Lipid storage involves the synthesis of nuclear lipid droplets from the INM and is characterized by lipid exchange through Seipin-dependent membrane bridges. We identify the genetic circuit for nuclear lipid droplet synthesis and a role of these organelles in regulating this circuit by sequestration of a transcription factor. Our findings suggest a link between INM metabolism and genome regulation and have potential relevance for human lipodystrophy.

## Highlights

• INM is metabolically active and stores lipids via nuclear lipid droplets (nLDs)
• Intranuclear lipid sensors detect DAG enrichment at INM and PA/DAG on nLDs
• Nutrients and Opi1 transcriptional circuit regulate nLD synthesis
• Lipodystrophy-related Seipin promotes formation of INM-nLD membrane bridges

## Graphical Abstract

**Figure:**
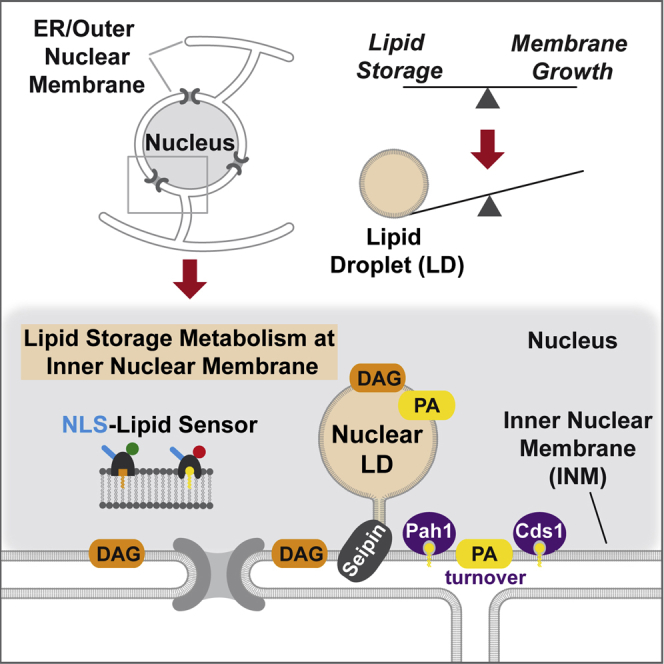

## Teaser

Active lipid metabolism and a distinct lipid composition of the inner nuclear membrane allow cells to produce nuclear lipid droplets.

## Introduction

**Figure 1:**
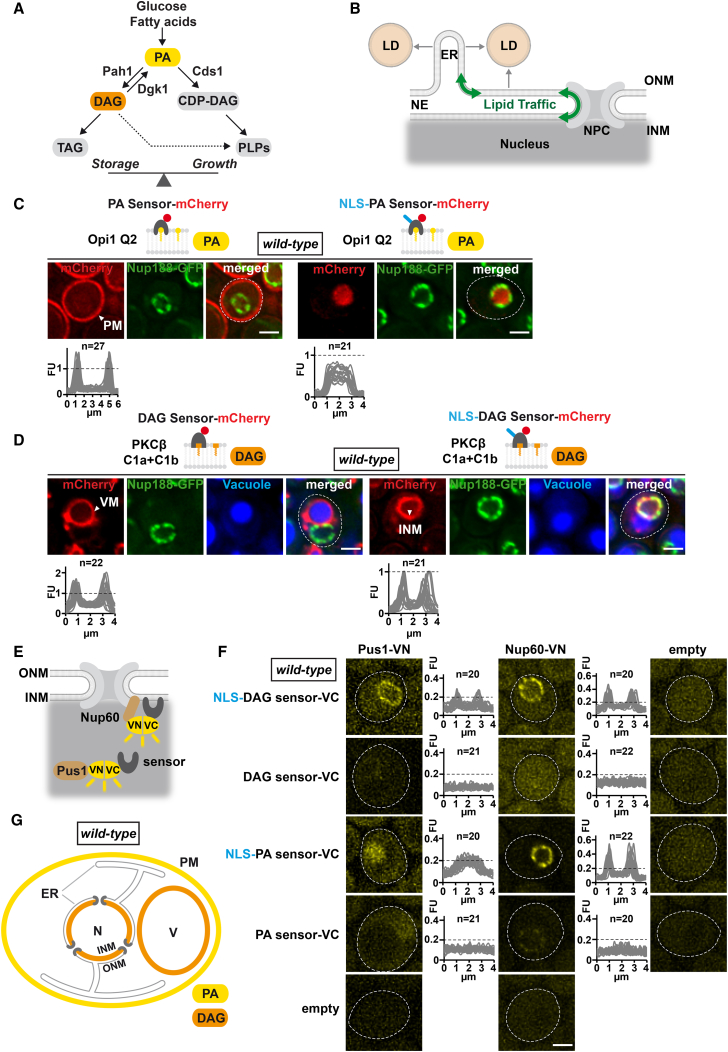
Lipid Biosensors for Probing the INM (A) Simplified cartoon of yeast lipid biosynthesis depicting the two major branches leading to synthesis of phospholipids (PLPs) (Growth) or triacylglycerol (TAG) (Storage). Phosphatidic acid (PA) is a central precursor. The Kennedy pathway (dashed line) channels diacylglycerol (DAG) into PLP production. CDP-DAG, cytidine diphosphate diacylglycerol. (B) Presumed lipid traffic between the contiguous membranes of endoplasmic reticulum (ER), outer nuclear membrane (ONM), and inner nuclear membrane (INM). Lipid droplets (LDs) form on ER and ONM. NPC, nuclear pore complex. (C) Live imaging of cells expressing the plasmid-based PA sensor Opi1 Q2-mCherry with or without an N-terminal Nup60 nuclear localization sequence (NLS). Nup188-GFP marks the nuclear envelope; dashed white line indicates the cell contour. Sensor fluorescence intensity was quantified across a line spanning the whole cell (left) or the nucleus (right). For comparison, the FU value 1 is marked with a horizontal dashed line. n = number of randomly selected cells. FU, arbitrary fluorescence units; PM, plasma membrane. Scale bar, 2 μm. (D) Live imaging of cells expressing the plasmid-based DAG-mCherry sensor with or without the N-terminal NLS. Vacuoles stained with CellTracker Blue. Sensor fluorescent intensity was quantified across the vacuole (left) or the nucleus (right). INM, inner nuclear membrane; VM, vacuolar membrane. Scale bar, 2 μm. (E) Experimental design for BiFC (bimolecular fluorescence complementation). VN, VC, complementary Venus fragments. (F) Live imaging of cells expressing the indicated BiFC constructs. Lipid sensors are fused with VC, Nup60, and Pus1 with VN. Empty vectors are used as controls. Fluorescent intensity was quantified across the nucleus. n = number of randomly selected cells. Scale bar, 2 μm. (G) Apparent localization of major PA and DAG pools in wild-type cells as detected by lipid biosensors. N, nucleus; V, vacuole; otherwise abbreviations are the same as above. See also Figure S1, Figure S2, Figure S3, Figure S7.

The endoplasmic reticulum (ER) synthesizes phospholipids for membrane growth and cell proliferation, and triacylglycerol (TAG) to store energy (van Meer et al., 2008). TAG and phospholipids have common precursors, and the flow of lipids into storage or growth is a key cellular decision that is balanced with nutrient availability (Figure 1A). Phosphatidic acid (PA) is a lipid precursor at the branch point of the lipid storage and growth pathways (Figure 1A) (Carman and Han, 2011). PA is either converted into cytidyldiphosphate diacylglycerol (CDP-DAG) by yeast Cds1 (human CDS1) to promote new phospholipid synthesis or is dephosphorylated by Pah1 (human Lipin) to produce diacylglycerol (DAG). DAG, a signaling lipid, is phosphorylated by the Dgk1 kinase to regenerate PA, or is metabolized further into TAG. TAGs and steryl esters are stored in lipid droplets, which are synthesized on the ER and outer nuclear membrane (ONM) (Hashemi and Goodman, 2015, Thiam et al., 2013). Although lipid droplets have sporadically been observed inside the nucleus of some mammalian cells (Layerenza et al., 2013, Ohsaki et al., 2016, Uzbekov and Roingeard, 2013), lipid droplet synthesis is considered to be the task of the ER/ONM (Figure 1B).

The lipid composition of cellular organelles is customized to their functions, and the lipid composition of membranes can vary, including lipid asymmetry across the bilayer as well as in the lateral dimension (van Meer et al., 2008). The ER, the cell’s main lipid factory, is contiguous with the ONM, which is joined with the inner nuclear membrane (INM) at membrane openings occupied by nuclear pore complexes (NPCs) (Ungricht and Kutay, 2017). Given that lipids can laterally diffuse within membranes (Figure 1B), it is generally assumed that the INM passively receives lipids from the ONM/ER and is itself metabolically silent (van Meer et al., 2008).

The INM is enriched in proteins that are required for genome regulation, inheritance, and protection, including integral and peripheral membrane proteins (Ungricht and Kutay, 2017). Recent evidence suggests that specific lipids interact with the transmembrane domain of particular proteins (Gao et al., 2016). Hence, the specific INM proteome might reflect a functionally tailored lipid environment. It is therefore important to know what the INM lipids are and where they come from.

We have developed tools to directly examine the INM lipid composition in living cells and combine a lipid biosensor strategy with specific perturbations of lipid metabolism. Our study argues that the INM is a metabolically active territory, whose chemical reactions could influence numerous aspects of genome function.

## Results

### Lipid Biosensors for Probing the INM

**Figure S1:**
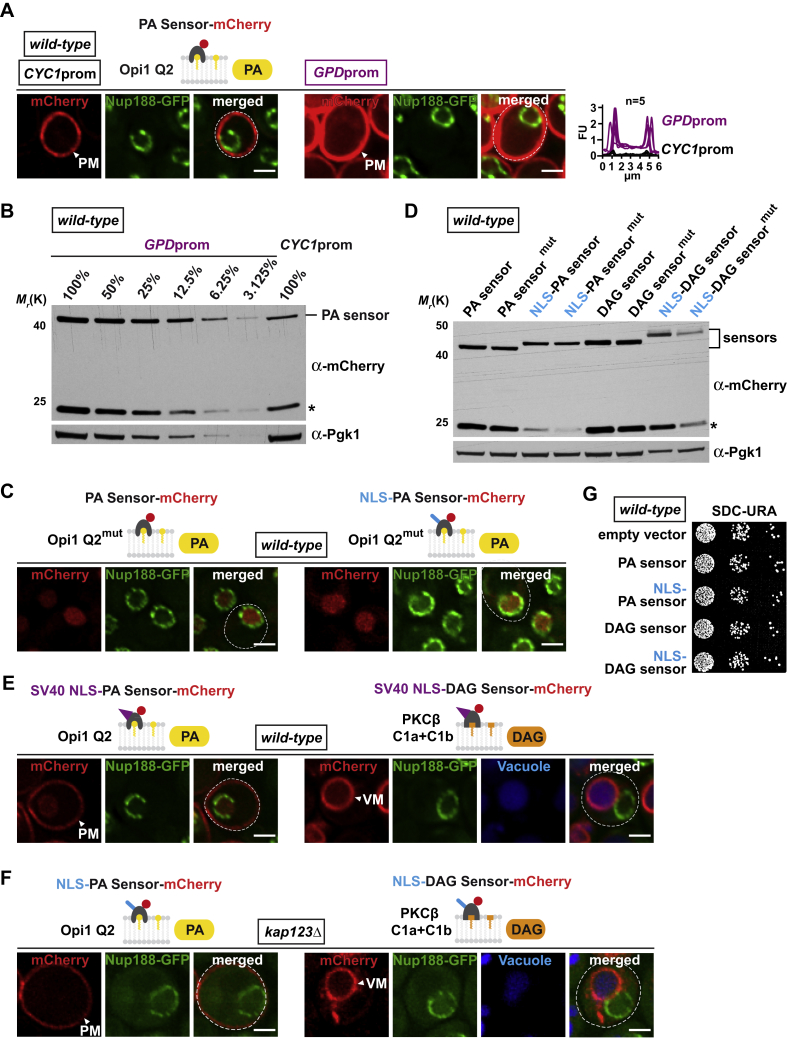
Characterization of Lipid Sensor Specificity and Nuclear Import, Related to Figure 1 (A) Overexpression of the Opi1 Q2 sensor detects the same cellular distribution of PA. Live imaging of exponentially growing cells expressing the plasmid-based PA sensor Opi1 Q2-mCherry under the *CYC1* or *GPD (TDH3)* promoter. Nup188-GFP labels NPCs. Images were taken with the same exposure time and scaling. Line-scan graphs generated in ImageJ quantify the increase in sensor fluorescent intensity at the PM upon overexpression. n indicates the number of randomly selected cells, y axis: Arbitrary Fluorescence Units, FU; x axis: distance in μm. Dashed line marks the cell contours. Plasma membrane, PM. Scale bar: 2 μm. (B) Comparison of PA sensor protein levels when expressed from the *CYC1* or stronger *GPD (TDH3)* promoter in wild-type cells. Denaturing extracts were prepared and immunoblotted with an anti-mCherry antibody directed against the sensors and with an anti-Pgk1 (3-phosphoglycerate kinase) antibody as a loading control. Serial dilutions of cell extracts are shown. Asterisk indicates mCherry-reactive degradation product. (C) Live imaging of cells expressing the indicated plasmid-based sensors and genomically integrated Nup188-GFP. Mutations in Opi1 Q2 (Q2^mut^) were previously characterized to reduce PA affinity as shown by the lack of PA detection at the plasma membrane (Loewen et al., 2004). The mutant PA sensor lacking an NLS is imported into the nucleus due to an endogenous NLS overlapping with the Q2 domain (Loewen et al., 2004). Scale bar: 2 μm. (D) Comparison of the indicated sensor protein levels in wild-type cells. Denaturing extracts were prepared and immunoblotted with an anti-mCherry antibody against the sensors and with an anti-Pgk1 antibody as a loading control. Asterisk indicates mCherry-reactive degradation product. (E) Live imaging of cells expressing the indicated plasmid-based PA and DAG sensors, both carrying an N-terminal Simian-Virus 40 large T-antigen nuclear localization sequence (SV40 NLS). This type of NLS is known to depend on the Kap60/Kap95 import pathway. Compared to the Nup60 NLS, the SV40 NLS failed to import the lipid sensors into the nucleus. Vacuoles are stained using CellTracker Blue. Plasma membrane, PM; vacuolar membrane, VM. Scale bar: 2 μm. (F) Kap123 is required for importing lipid sensors harboring an NLS present in aa1-24 of Nup60 (Mészáros et al., 2015). Live imaging of *kap123Δ* cells expressing a plasmid-based NLS-PA sensor or NLS-DAG sensor and genomically integrated Nup188-GFP. Both sensors exhibit the distribution of the non-NLS sensors upon inhibition of nuclear import. Thus, DAG and PA recognition is not generally impaired by the Nup60 NLS or the SV40 NLS (E). Vacuoles are stained using CellTracker Blue. Plasma membrane, PM; vacuolar membrane, VM. Scale bar: 2 μm. (G) Growth analysis of wild-type cells transformed with the indicated plasmids. Growth was followed on SDC-Ura plates. Cells were spotted onto plates in 10-fold serial dilutions and incubated for 2 days at 30°C.

**Figure S2:**
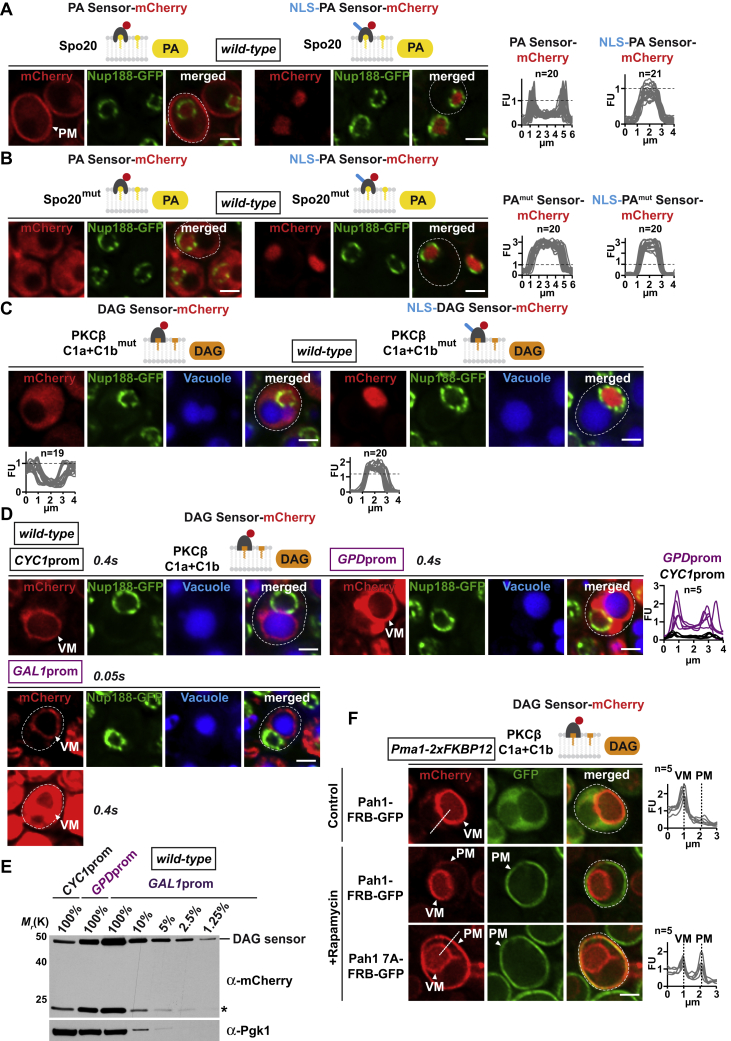
Characterization of Lipid Sensor Specificity, Related to Figure 1 (A) Live imaging of cells expressing the plasmid-based PA sensor Spo20-mCherry with or without the Nup60 NLS. Spo20 is a sporulation-specific protein required for the formation of the yeast prospore membrane (Nakanishi et al., 2004). The Spo20 sensor detects PA pools in the same subcellular localization as Opi1 Q2 with high PA levels at the plasma membrane and low PA levels at the INM. Line-scan graphs generated in ImageJ were used to quantify sensor fluorescent intensity at the PM and in the nucleus. n indicates the number of randomly selected cells, y axis: Arbitrary Fluorescence Units, FU; x axis: distance in μm. Plasma membrane, PM. Scale bar: 2 μm. (B) Specificity control for the Spo20 PA sensors. A mutant version of Spo20 (Nakanishi et al., 2004) no longer detected PA at the plasma membrane (left panel). Line-scan graphs were used to quantify sensor fluorescent intensity at the plasma membrane (line drawn across plasma membrane and cytoplasm) and in the nucleus. Scale bar: 2 μm. (C) Specificity controls for the indicated DAG sensors. The PKCβ C1a+C1b was mutated at residues Q63 and Q128, which are critical for DAG sensing (Lučić et al., 2016). The NLS-sensor no longer detected DAG at the INM (right panel), whereas the non-NLS sensor failed to detect DAG at the vacuole (left panel). See Figure S1D for protein expression/stability. Line-scans quantify sensor fluorescent intensity across the vacuole (line drawn across vacuole and cytoplasm) and in the nucleus. n indicates the number of randomly selected cells, y axis: Arbitrary Fluorescence Units, FU; x axis: distance in μm. Vacuoles are stained with CellTracker Blue. Scale bar: 2 μm. (D) ∼40-fold overexpression of the PKCβ C1a+C1b sensor does not detect DAG at the ONM or ER. Live imaging of exponentially growing cells expressing the plasmid-based DAG sensor C1a+C1b-mCherry under the *CYC1*, stronger *GPD* or highly inducible *GAL1* promoter. Nup188-GFP labels the nuclear envelope. The *GAL1* promoter causes high sensor overexpression and saturation of the maximum intensity values when recorded with the same imaging exposure as the *CYC1* or *GPD* promoter (0.4 s) and is therefore also recorded with 0.05 s. Line-scan graphs quantify the increase in sensor fluorescent intensity at the VM upon overexpression from the *GPD* promoter. n indicates the number of randomly selected cells, y axis: Arbitrary Fluorescence Units, FU; x axis: distance in μm. Vacuolar membrane, VM. Scale bar: 2 μm. (E) Comparison of DAG sensor protein levels when expressed from the *CYC1*, *GPD* or *GAL1* promoter. Denaturing cell extracts were prepared and immunoblotted with an anti-mCherry antibody directed against the sensors and with an anti-Pgk1 antibody as a loading control. Serial dilutions of cell extracts were prepared. The *GAL1* promoter increases the sensor protein levels approximately 40-fold compared to *CYC1*. Asterisk indicates mCherry-reactive degradation product. (F) Live imaging of Pma1-FKBP12 cells expressing the plasmid-based DAG sensor and Pah1-FRB-GFP or Pah1 7A-FRB-GFP. Cells were treated with a final concentration of 1 μg/mL of rapamycin for 1 hr. Targeting wild-type Pah1 to the PM resulted in a modest increase of DAG in this location, possibly because of an inefficient activation by the ER-resident Nem1-Spo7 phosphatase complex, which activates Pah1 through dephosphorylation (Santos-Rosa et al., 2005). A Pah1 variant, which harbors 7 Ser to Ala point mutations (Pah1 7A) at known Pah1 phosphorylation sites, is thought to bypass the need for Nem1-Spo7 dephosphorylation and is constitutively active (O’Hara et al., 2006). Accordingly, Pah1 7A strongly increased DAG levels at the PM. Line-scan graphs were used to compare sensor fluorescence intensity at the VM and the PM. Lines were drawn across these two membranes (3 μm dashed white lines) in cells with similar corrected total cell fluorescence (CTCF). Measurements confirm that the sensor can simultaneously recognize DAG at the vacuole and other endomembranes. This also excludes the possibility that sequestration of the sensor on the vacuole would prevent it from recognizing DAG at the ONM/ER consistent with sensor overexpression experiments (D). Line-scans were aligned with a similar VM-PM distance (vertical dashed lines). Vacuolar membrane, VM; plasma membrane, PM. Scale bar: 2 μm.

The INM and ONM are only 10–50 nm apart and biochemical fractionation fails to separate them into pure fractions that would be suitable for lipidomics (Monneron et al., 1972). In order to explore the lipid composition of the INM, we designed a set of genetically encoded, fluorescently labeled biosensors that visualize PA and DAG in living yeast cells. By appending or omitting a nuclear localization signal (NLS) to the sensors, we can assess PA and DAG levels in the nuclear as well as the cytoplasmic membrane compartment. The Q2 domain of the *S. cerevisiae* transcription factor Opi1 specifically recognizes high PA levels at the plasma membrane with a consistent pattern across a cell population (Figure 1C) confirming earlier reports (Loewen et al., 2004). When increasing the sensor concentration about 10-fold, the fluorescence intensity at the plasma membrane increases correspondingly, but no other membrane compartments become stained (Figures S1A and S1B). In contrast to this cytoplasmic sensor, an NLS version of the PA sensor showed a diffuse intranuclear signal (Figure 1C; see Figures S1C for sensor specificity, S1D for expression levels, and S1E and S1F for the import mechanism). Consistent results were obtained by using the PA-sensing domain of the *S. cerevisiae* Spo20 protein (Figures S2A and S2B) (Nakanishi et al., 2004). These data suggest that PA is present at lower levels at the INM and ONM/ER compared to the PA-rich plasma membrane under the conditions tested. To detect the downstream lipid DAG, we used the DAG-specific recognition domains of *R. norvegicus* protein kinase C (PKCβ C1a+C1b) (Lučić et al., 2016). We detected DAG predominantly at the vacuolar membrane, but not at the ONM and ER (Figure 1D; see also Figures S2C for sensor specificity and S1D for expression levels). This specific distribution was retained when we overexpressed the sensor (Figures S2D and S2E). Both 10-fold and approximately 40-fold overexpression strongly increased the signal at the vacuole, yet little DAG signal was observed at the ONM/ER or the plasma membrane. This suggests a major difference in DAG levels between the vacuolar membrane and the ONM/ER/plasma membrane. To test whether the sensor can in principle detect DAG in membrane compartments other than the vacuole, we conditionally targeted Pah1 to the PA-rich plasma membrane in order to ectopically convert PA into DAG. Upon tethering a constitutively active variant of Pah1 (Pah1 7A) to the plasma membrane protein Pma1, the DAG sensor stained the plasma membrane in addition to the vacuole, with about equal intensity (Figure S2F). This indicates that the DAG sensor is able to detect newly synthesized DAG at an ectopic location, and that enrichment of the sensor on the vacuole does not prevent it from recognizing other DAG-containing membranes.

Given the lack of DAG detection at the ONM/ER, it was striking to see that the NLS-bearing version of the DAG sensor targeted the INM (Figure 1D). The NLS-DAG sensor is expressed at similar levels as the non-NLS version and the sensor fluorescent intensities at the INM and vacuolar membrane are in a similar range (Figures 1D and S1D). This suggests that the difference in DAG detection between ONM/ER and INM is not a result of different sensor concentrations. Moreover, a version of the NLS-DAG sensor (Q63/Q128 > E) that is deficient in DAG binding distributes throughout the nucleoplasm (Figure S2C). Hence, the nuclear rim localization of the sensor is most likely a consequence of DAG recognition at the INM rather than exclusion by chromatin or unspecific interactions with INM proteins. Finally, DAG recognition per se does not appear to be influenced by appending an NLS to the sensor, since the NLS-DAG sensor was bound to the vacuole when its nuclear import was blocked (Figure S1F). In summary, our findings indicate a potential enrichment of DAG at the INM.

To validate the INM localization of the DAG sensor, we performed bimolecular fluorescence complementation (BiFC) with lipid sensors fused to the C-terminal half of the Venus fluorophore (VC) and two nuclear proteins, Pus1 and Nup60, fused to the N-terminal half of the fluorophore (VN) (Figure 1E). Pus1 is a pseudouridine synthase in the nucleoplasm; Nup60 is a basket nucleoporin, exclusively localized on the nuclear face of the NPC. The NLS version of the DAG sensor interacted with the nucleoplasmic Pus1-VN specifically at the nuclear rim consistent with the presence of DAG at the INM (Figure 1F). Also, Nup60 interacted with the NLS-DAG sensor confirming its INM localization. In contrast, the DAG sensor without NLS failed to interact with Pus1-VN and Nup60-VN. The NLS-PA sensor showed fluorescent complementation with Pus1-VN in the nucleoplasm and Nup60-VN at the nuclear rim consistent with its nucleoplasmic localization in wild-type cells (Figure 1F). A faint BiFC signal of the PA sensor without exogenous NLS in Nup60-VN-expressing cells likely stems from an intrinsic NLS that is present in the Q2 domain of the PA sensor and leads to weak nuclear import (Loewen et al., 2004). The PA and DAG sensors did not interfere with cell growth under the conditions tested, making it unlikely that they cause major alterations of lipid metabolism (Figure S1G).

**Figure S3:**
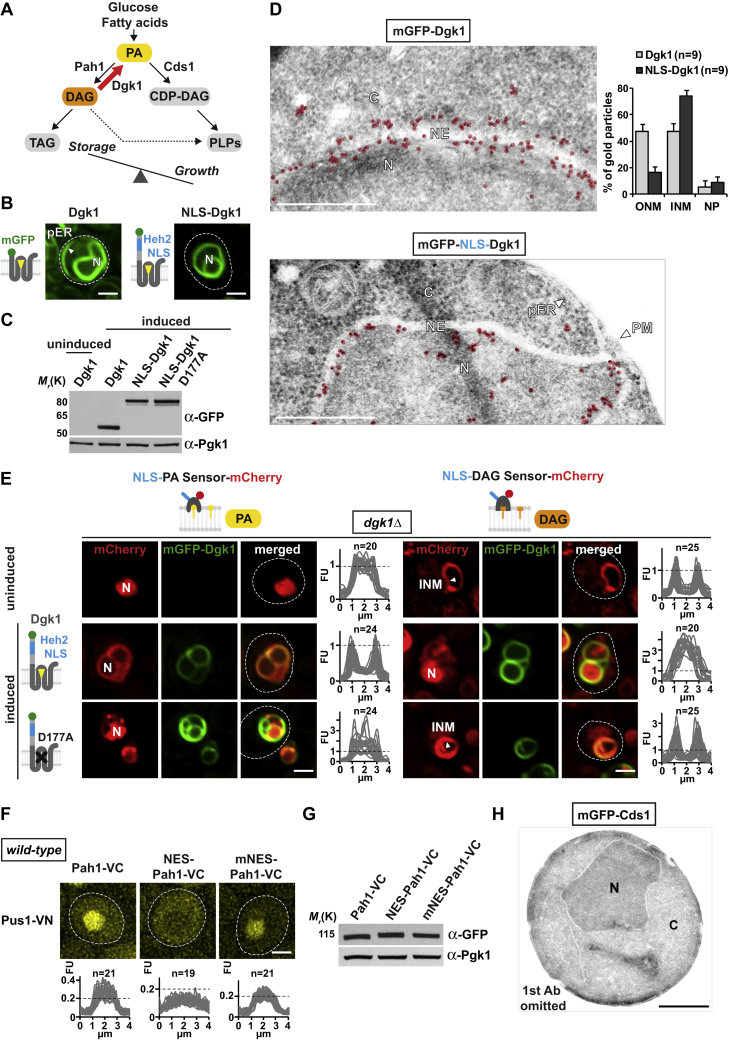
Increased Dgk1 Activity at the INM Modulates PA and DAG Levels, Related to Figures 1 and 3 (A) Cartoon depicts a predicted shift toward the growth branch of lipid metabolism upon Dgk1 overexpression (Henry et al., 2012). For abbreviations of lipid species, see Figure 1A. (B) Live imaging of *dgk1*Δ cells expressing plasmid-based wild-type Dgk1 or an NLS-Dgk1 construct, both N-terminally tagged with mGFP. The exogenous NLS sequence comprises the NLS of the INM transmembrane protein Heh2 and an adjacent linker (aa 93-317). The transmembrane protein Dgk1 was overexpressed from the inducible *GAL1* promoter. Cells were grown exponentially in raffinose-containing media and Dgk1 expression was induced with 2% galactose (final) for 4 hr before imaging. Dgk1 overexpression causes NE proliferation as shown by nuclear deformation and growth of additional NE structures, which are labeled by mGFP-Dgk1 (Han et al., 2008a). White arrowhead shows Dgk1 localization at the peripheral ER. Nucleus, N. Scale bar: 2 μm. (C) Comparison of protein levels of mGFP-tagged Dgk1 constructs when expressed from the *GAL1* promoter in *dgk1*Δ cells. Denaturing extracts were prepared and immunoblotted with an anti-GFP antibody and with an anti-Pgk1 antibody as a loading control. Induced refers to protein expression in the presence of 2% galactose. (D) Immunogold TEM of representative *dgk1Δ* cells expressing mGFP-tagged Dgk1 or NLS-Dgk1 constructs as in (B). Wild-type Dgk1 is found on both sides of the NE whereas NLS-Dgk1 is enriched on the INM side. Gold particles were false colored in transparent red. Gold particle quantification was performed by counting particles within a 125 nm zone relative to the NE midline (INM and ONM side) or in the nucleoplasm (NP) (> 125 nm from NE midline). Dgk1: 760 gold particles; NLS-Dgk1: 877 particles. n indicates number of analyzed nuclei, error bars indicate standard deviation. Nuclear envelope, NE; inner nuclear membrane, INM; outer nuclear membrane, ONM; nucleus, N; cytoplasm, C; peripheral endoplasmic reticulum, pER; plasma membrane, PM. Scale bar: 0.5 μm. (E) Live imaging of *dgk1Δ* cells expressing the plasmid-based NLS-PA sensor or NLS-DAG sensor and the indicated mGFP-Dgk1 constructs under the *GAL1* promoter. Cells were grown in raffinose containing media and Dgk1 expression was induced by addition of galactose (2% final) for 4 hr before imaging. The uninduced condition recapitulates the DAG and PA distribution seen in wild-type cells (compare with Figures 1C and 1D, right panels). Upon induction of NLS-Dgk1, the PA sensor detects increased PA levels at the INM (left panel), whereas the DAG sensor exhibits a nucleoplasmic location indicating reduced DAG levels (right panel). A catalytically inactive Dgk1 was created by mutating D177 > A, a conserved catalytic residue (Han et al., 2008b). As in wild-type cells this mutant exhibits a mainly nucleoplasmic location of the PA sensor and an INM location of the DAG sensor. Line-scan graphs generated in ImageJ were used to quantify sensor fluorescence intensity across the nucleus. n indicates the number of randomly selected cells, y axis: Arbitrary Fluorescence Units, FU; x axis: distance in μm. Note that overexpression of the catalytically inactive Dgk1 also causes some NE proliferation, which is likely caused by membrane stress, a phenomenon that may also result in NE ‘karmellae’ formation. Nucleus, N; inner nuclear membrane, INM. Scale bar: 2 μm. (F) Live imaging of cells expressing the indicated BiFC constructs. Wild-type Pah1, Pah1 with an exogenous nuclear export signal (NES, Rna1aa316-357) or Pah1 with a mutant NES (mNES) are fused with the Venus fragment VC; Pus1 is fused with the Venus fragment VN. The mNES contained the following mutations L320A, L323A, L326A, I328A, L340A, L342A according to (Feng et al., 1999). Line-scan graphs were used to quantify BiFC signals across multiple nuclei. The same FU value is marked for comparison (horizontal dashed line), n = number of randomly selected cells. Scale bar: 2 μm. (G) Comparison of protein levels of Pah1 constructs fused with the Venus fragment VC. Denaturing extracts were prepared and immunoblotted with an anti-GFP antibody (capable of detecting the Venus fragment VC) and with an anti-Pgk1 antibody as a loading control. (H) Immunogold TEM control sample of wild-type cells expressing mGFP-Cds1, in which the primary antibody was omitted. Sample shows no unspecific staining by the secondary antibody (anti-rabbit IgG coupled with 6 nm gold). The same outcome was observed for the Dgk1 and Pah1 samples analyzed in this study (not shown). Nucleus, N; cytoplasm, C. Scale bar: 1 μm.

If DAG is indeed enriched at the INM, we should be able to convert this pool into PA by experimentally increasing Dgk1 activity at the INM (Figure S3A). Dgk1 is an ER-resident transmembrane protein and is expected to follow a different nuclear import route than soluble cargo. We therefore appended the NLS of the INM-resident transmembrane protein Heh2 (Meinema et al., 2011) to Dgk1 and analyzed its localization. Overexpressed wild-type Dgk1 was detected both at the peripheral ER and at the nuclear envelope (NE) and induced NE proliferations consistent with previous reports (Figures S3B and S3C) (Han et al., 2008a). In contrast, the NLS version of Dgk1 removed the enzyme from the peripheral ER while targeting the NE, suggesting its nuclear import (Figure S3B). Indeed, immunogold electron microscopy (EM) demonstrated that wild-type Dgk1 is present on both sides of the NE (i.e., INM and ONM), whereas NLS-Dgk1 is enriched mainly at the inner side of the NE (Figure S3D). Importantly, NLS-Dgk1 caused a release of the NLS-DAG sensor from the INM into the nucleoplasm with concomitant binding of the NLS-PA sensor to the INM, whereas a catalytically inactive mutant of NLS-Dgk1 failed to release the NLS-DAG sensor from the INM (Figure S3E). These results suggest that our lipid sensors can specifically detect a Dgk1-mediated interconversion of DAG into PA at the INM.

Taken together, our data demonstrate that a DAG lipid sensor recognizes the INM differently from the ONM/ER, likely as the result of a different lipid composition (Figure 1G). This raises the important question whether the INM exhibits a unique lipid metabolic activity, which could be functionally important in cell physiology.

### Cds1 and Nutrients Regulate Nuclear Lipid Droplet Synthesis at the INM

**Figure 2:**
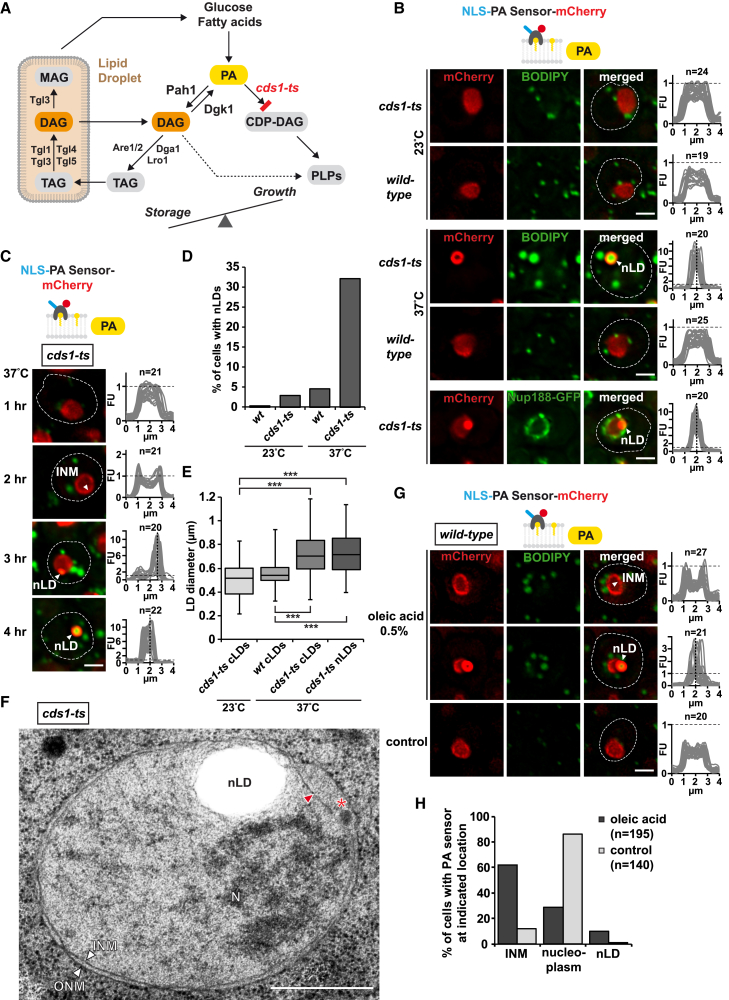
Cds1 and Nutrients Regulate Nuclear Lipid Droplet Synthesis at the INM (A) Cartoon depicts a shift toward the storage branch of lipid metabolism (Henry et al., 2012) through Cds1 inactivation *(cds1-ts)*. TAG (triacylglycerol) is stored in lipid droplets, which are metabolized by specific enzymes. MAG, monoacylglycerol; other abbreviations are as in Figure 1A. (B) Live imaging of wild-type or *cds1-ts* cells expressing the NLS-PA-mCherry sensor grown at the indicated temperatures for 4 hr. BODIPY stains lipid droplets; Nup188-GFP visualizes nuclear pores. Sensor fluorescent intensity was quantified across the nucleus as in Figure 1. The line scan was centered on nuclear lipid droplets (nLD) when present (dashed vertical line). Scale bar, 2 μm. (C) Time course of nLD formation in *cds1-ts* cells expressing NLS-PA-mCherry sensor. Exponentially growing cells were shifted from 23°C to 37°C and examined at the indicated time points. See also Videos S1 and S2. Scale bar, 2 μm. (D) Quantification of cells with nLDs. Cells were grown for 4 hr at the indicated temperatures. nLDs were defined as spherical structures that stained both with BODIPY and NLS-PA-mCherry sensor. At least 200 cells were counted for each condition. (E) Comparison of nuclear and cytoplasmic lipid droplet diameter in cells grown at the indicated temperatures. Box-whisker plot showing median, interquartile range, and minimum and maximum value. ^∗∗∗^p value <0.001 determined by ANOVA with post hoc Tukey HSD. (F) Transmission electron microscopy (TEM) of a representative *cds1-ts* cell after growth at 37°C for 4 hr. An nLD localizes next to the INM. The lumen of the NE is widened in a discrete portion (red arrowhead) and contains electron-dense material (red asterisk). Abbreviations are as before. Scale bar, 0.5 μm. (G) Live imaging of wild-type cells grown in oleic-acid-containing or control media, expressing the NLS-PA-mCherry sensor and stained with BODIPY. Two representative phenotypes of oleic-acid-treated cells are shown. Fluorescent intensity was quantified across the nucleus as in (B). Scale bar, 2 μm. (H) Quantification of NLS-PA sensor localization as observed in (G). n = number of analyzed cells. See also Figure S4 and Videos S1 and S2.

**Figure S4:**
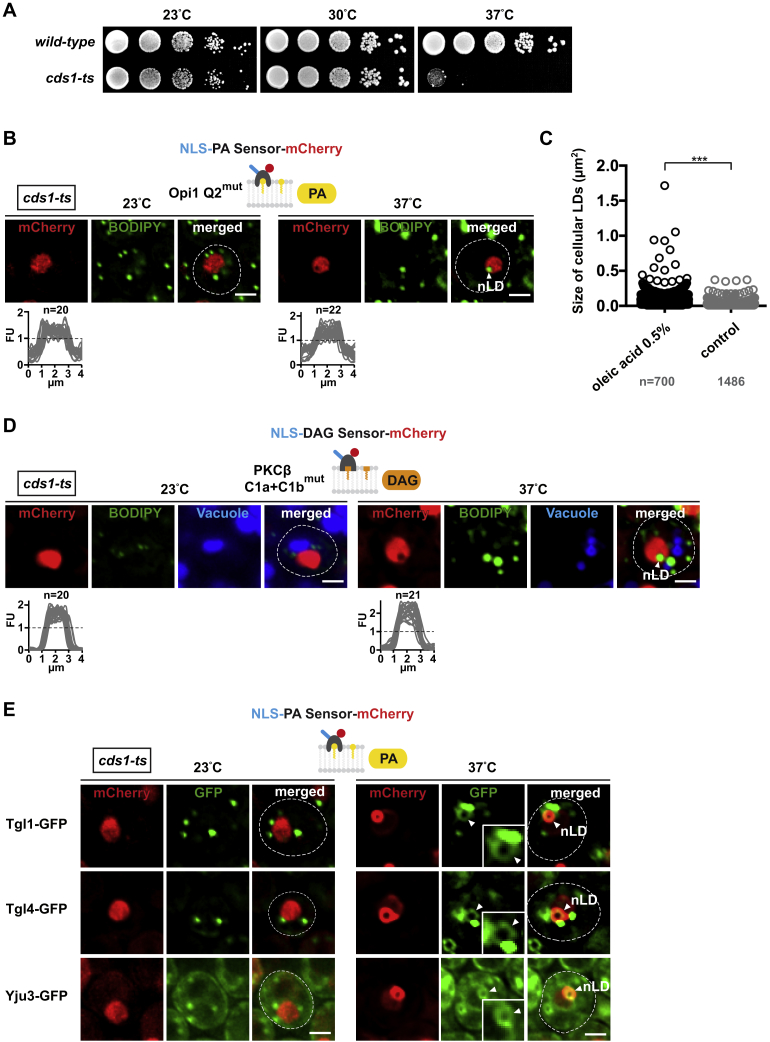
DAG, PA, and Enzymes of Lipid Metabolism Are Enriched on the nLD Surface, Related to Figures 2 and 3 (A) Growth comparison of wild-type and *cds1-ts* cells at the indicated temperatures. Cells were spotted onto YPD plates in 10-fold serial dilutions and incubated for 2 days at indicated temperatures. (B) Live imaging of *cds1-ts* cells expressing a plasmid-based NLS-PA sensor mutated in residues critical for PA binding. The mutant sensor fails to recognize PA at the INM or on nuclear lipid droplets. Cells were grown at the indicated temperatures for 4 hours and co-stained with BODIPY to visualize lipid droplets. Line-scans were used to quantify sensor fluorescent intensity across the nucleus. n indicates the number of randomly selected cells, y axis: Arbitrary Fluorescence Units, FU; x axis: distance in μm. For comparison the FU value 1 is marked with a horizontal dashed line. Nuclear lipid droplet, nLD. Scale bar: 2 μm. (C) Automated quantification of lipid droplet size in wild-type cells upon oleic acid treatment. After setting identical fluorescence intensity thresholds, circular BODIPY structures were automatically selected and quantified using ImageJ. Number of analyzed cells is indicated. p value (^∗∗∗^ < 0.001) was determined by Wilcoxon signed-rank test. (D) Live imaging of *cds1-ts* cells expressing a plasmid-based NLS-DAG sensor mutated in residues critical for DAG recognition. The DAG pool at the INM and nLDs is no longer recognized as shown by the nucleoplasmic sensor signal. Cells were grown at the indicated temperatures for 4 hours and co-stained with BODIPY to visualize lipid droplets and CellTracker Blue for vacuoles. Line-scans were used to quantify sensor fluorescent intensity across the nucleus. n indicates the number of randomly selected cells, y axis: Arbitrary Fluorescence Units, FU; x axis: distance in μm. For comparison the FU value 1 is marked with a horizontal dashed line. Nuclear lipid droplet, nLD. Scale bar: 2 μm. (E) Live imaging of *cds1-ts* cells expressing GFP-tagged enzymes previously implicated in cytoplasmic lipid droplet metabolism. Cells co-express a plasmid-based NLS-bearing PA-mCherry sensor to visualize nLDs. Cells were grown for 4 hours at the indicated temperatures. Arrowheads highlight enzymes that co-localize with the PA-rich nLD monolayer. Other GFP-foci or circular structures reflect the association with cytoplasmic LDs. Inset shows magnified views of nLDs. Nuclear lipid droplet, nLD. Scale bar: 2 μm.

To explore whether the INM is metabolically active, we measured its lipid composition upon reprogramming lipid metabolism toward lipid storage (Figure 2A). Cds1 inhibition is expected to channel PA into the Pah1-dependent branch of TAG synthesis and storage. To create this condition, we employed a temperature-sensitive mutant of Cds1 (*cds1-ts*; Figure S4A) and measured PA levels at the INM with the NLS-bearing PA sensor at the permissive (23°C) and restrictive (37°C) temperatures. Lipid storage was monitored by staining lipid droplet (LD) neutral lipids with the BODIPY 493/503 dye. When *cds1-ts* cells were grown at the permissive temperature (23°C), they showed low PA levels at the INM and small cytoplasmic lipid droplets (cLDs), similar to wild-type cells grown at 23°C or 37°C (Figure 2B). In contrast, *cds1-ts* cells at the restrictive temperature (37°C) showed large cLDs, consistent with a role of yeast and human Cds1 in influencing lipid droplet size (Fei et al., 2011, Qi et al., 2016). Strikingly, we detected some large BODIPY-positive structures, typically one or two per cell, that enriched the NLS-bearing PA sensor on their surface. Moreover, these structures localized to the nuclear interior as judged by the NPC marker Nup188-GFP (Figure 2B; see Figure S4B for specificity control). These features suggest that the observed structures are intranuclear and represent nuclear lipid droplets (nLDs) with a core of BODIPY-stainable neutral lipids and a PA-rich lipid layer on the surface. To understand how nLDs form, we followed their emergence after Cds1 inhibition (Figure 2C and Videos S1 and S2). PA levels at the INM began to rise after 2 hr at the restrictive temperature, PA started to accumulate at small BODIPY-positive foci at the INM after 3 hr, and prominent nLDs had formed after 4 hr, which absorbed almost the entire pool of nuclear PA on their surface. Quantification of nLD biogenesis in multiple cells confirms the redistribution of the NLS-PA sensor from the nucleoplasm to the INM and, finally, onto the nLD surface. nLDs were found in approximately one-third of *cds1-ts* cells at 37°C (Figure 2D) and exhibited a similar diameter as cLDs (Figure 2E). To study nLD ultrastructure, we employed transmission electron microscopy (TEM). After 4 hr of Cds1 inactivation at 37°C, we observed prominent nLDs, often close to the INM (Figure 2F), which likely correspond to the BODIPY-positive and NLS-PA-sensor-enriched structures seen by live-cell fluorescence microscopy. To exclude the possibility that nLD production merely reflects a mutant condition, we supplemented exponentially growing wild-type cells with 0.5% oleic acid, a monounsaturated fatty acid, which cells can utilize as their sole carbon source. This high-fat diet induced an increase in cellular lipid droplet size (Figure S4C). It also resulted in a PA increase at the INM in around 60% of cells and led to nLD formation in 10% of cells as detected by simultaneous analysis with the NLS-PA sensor and BODIPY (Figures 2G and 2H). Thus, nLD formation in *cds1-ts* mutant cells reflects the natural capacity of cells to synthesize nLDs besides the known pool of cLDs. These findings also suggest that the conserved Cds1 enzyme limits both PA abundance at the INM and nLD synthesis from the INM.

Video S1. Biogenesis of Nuclear Lipid Droplets in *cds1-ts* Cells, Example 1, Related to Figure 2 Live imaging of *cds1-ts* cells expressing the NLS-bearing PA-mCherry sensor and co-stained with BODIPY to visualize lipid droplets. Cells were grown exponentially at the permissive temperature (23°C) and then transferred onto a μ-Slide in a temperature-controlled microscope chamber kept at the restrictive (37°C) temperature. Imaging was started after 40 min at 37°C and images were taken every 20 min for 4 hours. Scale bar: 2 μm.

Video S2. Biogenesis of Nuclear Lipid Droplets in cds1-ts Cells, Example 2, Related to Figure 2 Same as Video S1, but images were taken every 20 min for 3 hours. Scale bar: 2μm.

### Enzymes of Lipid Metabolism Are Targeted to the INM

**Figure 3:**
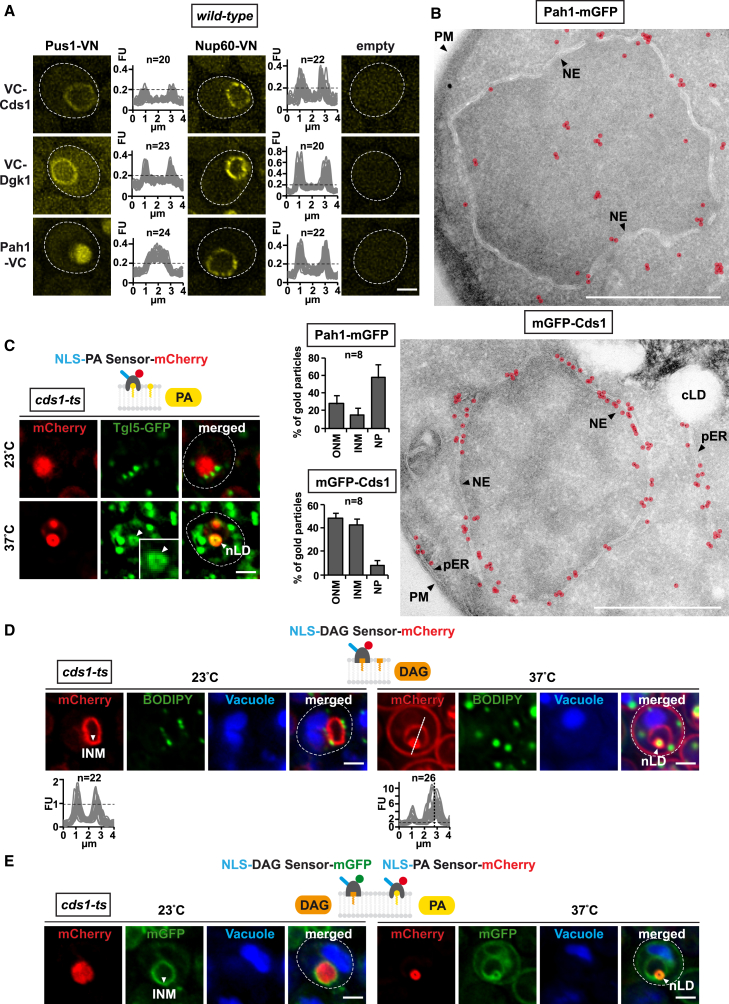
Enzymes of Lipid Metabolism Are Targeted to the INM (A) BiFC experiment with cells expressing the indicated enzymes fused with the Venus fragment VC, Nup60, or Pus1 are fused with VN. Empty vectors are used as controls. Fluorescent intensity was quantified across the nucleus as in Figure 1. n = number of randomly selected cells. Scale bar, 2 μm. (B) Immunogold TEM of representative wild-type cells expressing Pah1-mGFP or mGFP-Cds1. Gold particles are false colored in transparent red. Particles were counted within a 125-nm zone relative to the NE midline (INM and ONM side) or in the nucleoplasm (NP) (>125 nm from NE midline). Error bars: SD. n = number of analyzed nuclei. Pah1: 381 gold particles; Cds1: 602 particles. pER, peripheral endoplasmic reticulum; cLD, cytoplasmic lipid droplet; other abbreviations are as before. Scale bar, 1 μm. (C) Live imaging of *cds1-ts* cells expressing genomically integrated Tgl5-GFP and the plasmid-based NLS-PA-mCherry sensor. Cells were grown for 4 hr at the indicated temperatures. Inset shows magnified nLD; white arrowheads label the nLD surface. Scale bar, 2 μm. (D) Live imaging of *cds1-ts* cells expressing the NLS-DAG-mCherry sensor and stained with BODIPY for lipid droplets and CellTracker Blue for vacuoles. Cells were grown for 4 hr at the indicated temperatures. Sensor fluorescent intensity was quantified across the nucleus. Measurements from nLD-containing nuclei were aligned by nLD peak intensity (dashed vertical line). n = number of randomly selected cells. Scale bar, 2 μm. (E) Live imaging of *cds1-ts* cells co-expressing the NLS-DAG-mGFP and NLS-PA-mCherry sensor. Vacuoles were stained with CellTracker Blue. Cells were grown for 4 hr at the indicated temperatures. Scale bar, 2 μm. See also Figures S3 and S4.

Our results raised the question whether cells target specific lipid biosynthetic enzymes, such as Cds1, to the INM. To test this, we analyzed by BiFC whether key lipid enzymes, Cds1, Pah1, and Dgk1 (Figure 1A), localize inside the nucleus besides their known presence at the ER. Notably, BiFC detected all enzymes at the nucleoplasmic face of the NPC via Nup60-VN indicating an INM localization (Figure 3A). Pah1 additionally appeared in the nucleoplasm, as detected by Pus1-VN. The interaction pattern is consistent with Cds1 and Dgk1 being predicted transmembrane proteins, whereas Pah1 dynamically interacts with membranes via an amphipathic helix (Karanasios et al., 2010). The BiFC data on Dgk1 further confirm the immunogold localization of Dgk1 at both the ONM and INM (Figure S3D). Since Pah1 seemed to have a soluble pool in the nucleoplasm, we tested whether appending an exogenous nuclear export signal (Rna1 NES) would prevent its natural import into the nucleus. Indeed, the NES version of Pah1 caused a loss of BiFC signal, whereas a mutant NES restored BiFC with Pus1-VN (Figures S3F and S3G). To confirm these findings, we localized Pah1 and Cds1 by immunogold EM. Consistent with the BiFC data, Pah1 localized to the nucleoplasm with some gold labeling present at the NE (Figure 3B). Similar to Dgk1 (Figure S3D), Cds1 was detected on both sides of the NE but not in the nucleoplasm (Figures 3B and S3H). To further test whether nLDs could be regulated by characteristic lipid droplet enzymes involved in lipid storage metabolism, we tested whether Tgl5, Tgl4, Tgl1, and Yju3, a group of lipases involved in cLD turnover (Henry et al., 2012), co-localized with nLDs. Indeed, after *cds1-ts* inactivation, GFP-tagged versions of these enzymes localized to nLDs (Figures 3C and S4E). In sum, the localization of key lipid enzymes inside the nucleus indicates that the INM is a metabolically active lipid territory capable of generating nLDs, the end product of cellular lipid storage.

### PA and DAG Are Co-enriched on the Nuclear Lipid Droplet Surface

PA and DAG are both lipids that can induce membrane curvature. This property is thought to be important for the budding of cLDs from the ER as well as the fusion of individual lipid droplets, processes that involve the bending of membranes (Thiam et al., 2013). Since DAG is enriched at the INM (Figure 3D) and produced from PA, we sought to investigate whether DAG is also present on nLDs. Indeed, the nuclear NLS-DAG sensor enriched on nLDs upon Cds1 inhibition, while maintaining its localization at the INM (Figure 3D; specificity control in Figure S4D). To simultaneously monitor PA and DAG at the INM during nLD biogenesis, we co-expressed the NLS-DAG and NLS-PA sensors, tagged with different fluorophores in *cds1-ts* cells. At the permissive temperature, these sensors recapitulate the distribution of PA and DAG in wild-type cells (Figure 3E). At the restrictive temperature both sensors co-localized to ring-shaped structures that are characteristic of the nLD surface (Figure 3E). In sum, Cds1 has a major impact on the distribution of both PA and DAG at the INM and their co-enrichment on nLDs. This raises the question, by which mechanism these lipids are channeled to nLDs.

### Nuclear Lipid Droplets Are Generated Directly from the INM

**Figure 4:**
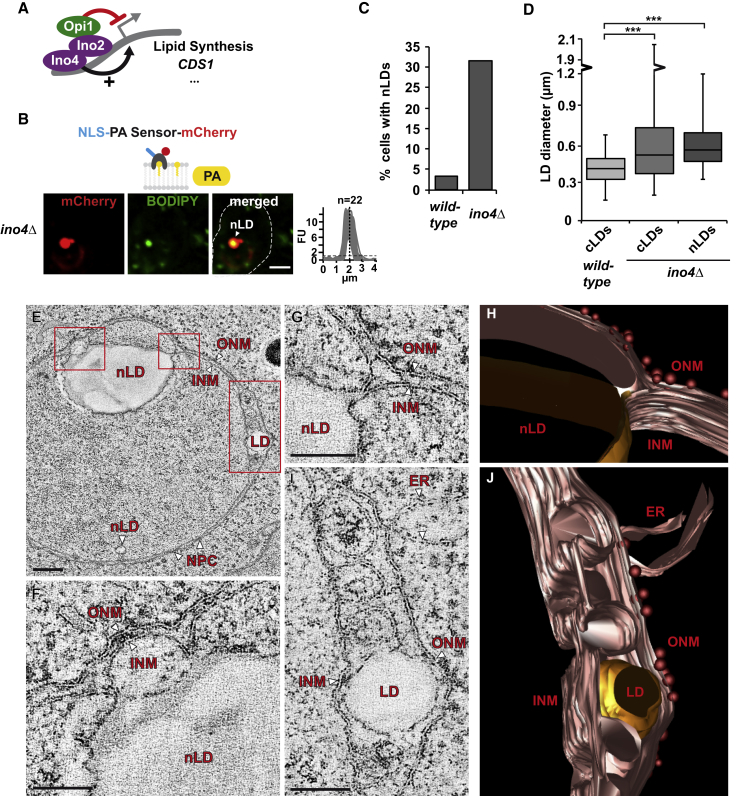
Nuclear Lipid Droplets Are Generated Directly from the INM (A) Cartoon of lipid synthesis control by the Ino2/Ino4 transcriptional activator and the Opi1 transcriptional repressor. *CDS1* and several other genes involved in phospholipid synthesis (e.g., *INO1*, *CHO1*, *CHO2*, *OPI3*, *PSD1*) are controlled by these factors. (B) Live imaging of *ino4Δ* cells expressing the NLS-PA-mCherry sensor. Lipid droplets are stained with BODIPY. Sensor fluorescent intensity was quantified across the nucleus as in Figure 2B. n = number of randomly selected cells. Scale bar, 2 μm. (C) Quantification of cells with nLDs; 150 wild-type and 400 *ino4Δ* cells were analyzed. (D) Comparison of nuclear and cytoplasmic lipid droplet diameter in the indicated strain backgrounds. Box-whisker plot showing median, interquartile range, and minimum and maximum value. ^∗∗∗^p value <0.001 was determined by Wilcoxon signed-rank test. (E–J) Transmission electron microscopy (TEM) and 3D reconstruction of the nuclear envelope in *ino4Δ* cells. (F) and (G) correspond to the boxed areas in the upper part of (E) and show INM-nLD membrane bridges. (I) shows a magnification of INM evaginations seen on the right side of (E). (H) and (J) are 3D reconstructions of (G) and (I), respectively. The ONM is studded with ribosomes (red spheres). See Video S3 for an animated 3D model. Scale bar, 200 nm (E) or 100 nm for images (F), (G), and (I). See also Figure S5 and Video S3.

**Figure S5:**
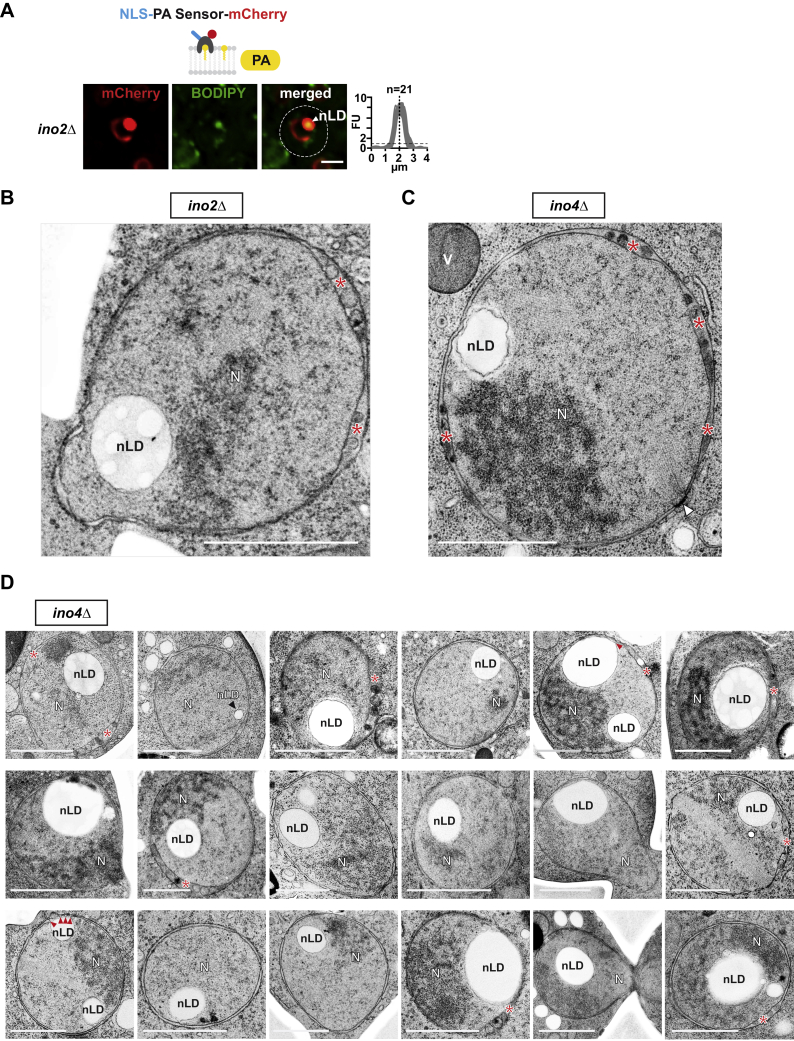
Disruption of Ino2/Ino4 Complex Results in nLD Formation, Related to Figure 4 (A) Live imaging of *ino2Δ* cells expressing the plasmid-based NLS-bearing PA-mCherry sensor. Lipid droplets were co-stained with BODIPY. Line-scan graphs quantify the fluorescent intensity across the nucleus. nLDs were aligned in the middle of the line scan (dashed vertical line). n indicates the number of randomly selected cells, y axis: Arbitrary Fluorescence Units, FU; x axis: distance in μm. For comparison with Figure 2B the FU value 1 is marked with a horizontal dashed line. Nuclear lipid droplet, nLD. Scale bar: 2 μm. (B) TEM analysis of *ino2Δ* cells reveals ultrastructure of a nuclear lipid droplet (nLD) and INM evaginations (asterisks). Nucleus, N. Scale bar: 1 μm. (C and D) TEM analysis of representative examples of *ino4Δ* cells. Note that multiple perinuclear zones with INM evaginations/cavities can occur (red asterisks). Membrane bridges that connect nLDs to the INM are indicated with red arrowheads. Nuclear lipid droplet, nLD; nucleus, N; vacuole, V. White arrowhead - spindle pole body. Scale bar: 1 μm.

The nLD-producing *cds1-ts* strain exhibited a drastic growth defect at the restrictive temperature (Figure S4A), which led us to search for mutants that allow a structural analysis of nLDs at standard growth temperature. We hypothesized that *INO4* and *INO2* deletion would phenocopy Cds1 inactivation because *CDS1* expression is promoted by the Ino4/Ino2 transcriptional complex (Figure 4A) (Carman and Han, 2011). Indeed, *ino4Δ* and *ino2Δ* cells displayed enlarged cLDs, as well as prominent nLDs with the characteristic NLS-PA-sensor enrichment at 30°C (Figures 4B and S5A). nLDs were present in approximately 1/3 of *ino4Δ* cells (Figures 4C and S5D) and had a similar size as the enlarged cLDs (Figure 4D). We used this condition to further characterize the relationship between nLDs and the INM by TEM. Interestingly, nLDs were found to interact with the INM at multiple membrane bridges (Figure 4E). By TEM, we could directly visualize the two opposing leaflets of the lipid bilayer and found that the lipid monolayer surrounding the nLD is contiguous with one of the two leaflets of the INM (Figures 4F and 4G). This suggests that PA, DAG, and other lipids can move from the INM onto the monolayer surrounding the nLD and into its interior. We reconstructed the 3D architecture of the INM-nLD contact points using stacks of electron micrographs (Video S3). The INM-nLD contacts do not form a stalk-like structure with a circular base but rather consist of an extended membrane ridge (Figure 4H). This membrane geometry probably requires scaffold proteins to be formed. Seipin is a possible candidate since it is implicated in stabilizing cLD-ER contacts (Grippa et al., 2015). A second feature of nLD-containing nuclei is the altered perinuclear space, with multiple evaginations of the INM, which measure about 70 nm at the membrane opening (Figures 4E, 4I, 4J, S5C, and S5D). INM evaginations are also abundant in *ino2Δ* cells (Figure S5B). Since lipid droplets are sometimes found in areas occupied by INM evaginations (Figures 4I and 4J), it is possible that these zones represent intermediate stages of nLD production. Multiple small nLDs that arise from INM evaginations may subsequently fuse with a larger nLD that remains tethered to the INM. In sum, we show that the INM directly forms nLDs and thereby contributes to cellular lipid storage.

Video S3. 3D Ultrastructural Analysis of an nLD-Containing Nucleus, Related to Figure 4 Electron tomography was performed on a 150 nm resin section derived from *ino4Δ* cells. The sample was tilted around two axes from −60° to +60°, each with a 1° increment. 3D animation shows a z-scan through a tomogram (1z = 2,2796 nm) and a model based on the ultrastructural contours of nuclear membranes. NE/ER membranes are labeled in bronze, lipid droplets in gold and ribosomes as red spheres. 3D animation corresponds to Figure 4E.

### Seipin Regulates the Formation of INM-nLD Membrane Bridges

**Figure 5:**
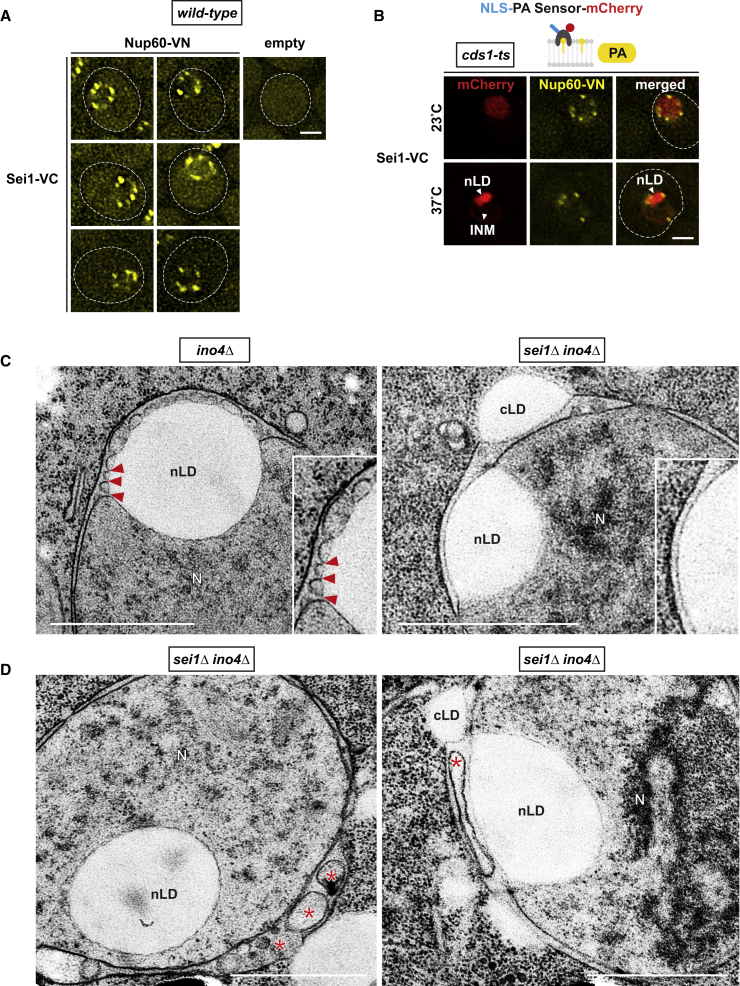
Seipin Regulates Formation of INM-nLD Membrane Bridges (A) Live imaging of representative cells expressing the indicated BiFC constructs. Empty vector co-expressed with Sei1-VC is used as a control. Scale bar, 2 μm. (B) Live imaging of *cds1-ts* cells expressing the NLS-PA-mCherry sensor and BiFC constructs. Cells were grown for 4 hr at the indicated temperatures. Scale bar, 2 μm. (C) Representative TEM images of *ino4Δ* cells and *sei1Δ ino4Δ* cells. An nLD is connected to the INM via numerous membrane bridges (red arrowheads) in the *ino4Δ* cell, which are absent in the *sei1Δ ino4Δ* mutant. Scale bar, 1 μm. (D) INM evaginations in the periplasmic space have highly irregular shapes and sizes (red asterisks) in *sei1Δ ino4Δ* cells (compare with Figure 4I). Scale bar, 1 μm. See also Figure S6.

For mammalian cytoplasmic lipid droplets, Seipin is thought to mediate lipid droplet maturation at the ER (Wang et al., 2016) and both PA and DAG were implicated in this process (Adeyo et al., 2011, Cartwright et al., 2015, Grippa et al., 2015, Karanasios et al., 2013, Wolinski et al., 2015). Mutations in Seipin lead to a severe form of congenital lipodystrophy, characterized by a lack of adipose tissue (Szymanski et al., 2007). Seipin, a predicted transmembrane protein, is conserved from yeast (*SEI1*) to human and localizes to the ER. In yeast, *SEI1* deletion results in clusters of irregularly sized lipid droplets (Szymanski et al., 2007). To test whether Seipin plays a role in nLD production, we first examined whether it is present at the INM. By performing BiFC analysis with Nup60-VN and Sei1-VC, we observed BiFC puncta (Figure 5A), which indicate the presence of Sei1 at the INM besides its known localization at the ER. Notably, after induction of nLDs by inactivation of Cds1, fluorescent puncta resulting from Sei1-VC interaction with Nup60-VN were found in the vicinity of nLDs (Figure 5B).

**Figure S6:**
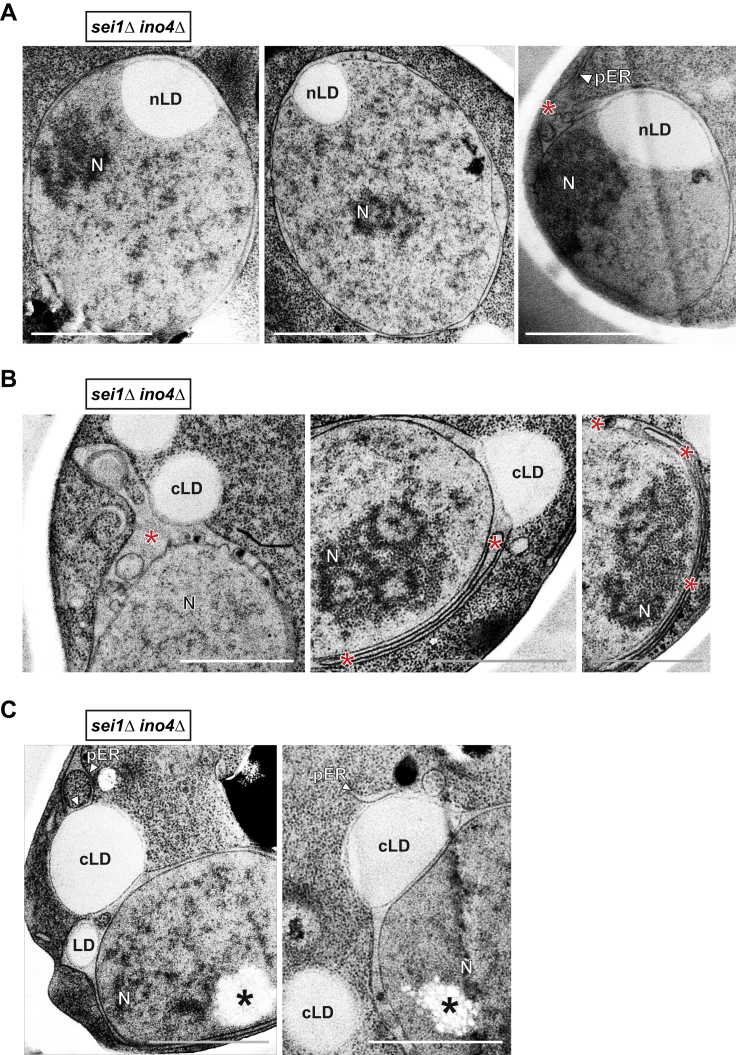
Seipin Regulates Formation of INM-nLD Membrane Bridges and Architecture of the Perinuclear Space, Related to Figure 5 (A) TEM analysis of *sei1Δ ino4Δ* cells. nLDs were found to adhere tightly to the INM. Irregular periplasmic spaces are indicated (red asterisk). Nuclear lipid droplet, nLD; nucleus, N; peripheral endoplasmic reticulum, pER. Scale bar: 1 μm. (B) Representative examples of periplasmic space abnormalities in *sei1Δ ino4Δ* cells. These can range from heterogeneously sized evaginations to large flat cavities (both marked with red asterisks). Cytoplasmic lipid droplet, cLD; nucleus, N. Scale bar: 1 μm. (C) TEM analysis of *sei1Δ ino4Δ* cells. cLDs also adhere to the ONM and/or pER. Clusters of small, aggregated LDs are indicated (black asterisks). Cytoplasmic lipid droplet, cLD; nucleus, N; peripheral endoplasmic reticulum, pER. Scale bar: 1 μm.

To directly examine a potential effect of Seipin on nLD formation, we deleted *SEI1* in *ino4Δ* cells (Figure 5C). Notably, we did not observe defined membrane bridges upon *SEI1* deletion. Instead, the nLDs were found to adhere closely to the INM (Figures 5C, S6A, and S6C). Whereas *ino4Δ* cells exhibited regularly sized evaginations in the periplasmic nuclear space (Figure 4I), *sei1Δ ino4Δ* cells displayed irregular periplasmic cavities (Figures 5D and S6B). In sum, we demonstrate that yeast Seipin, a key lipid droplet biogenesis factor, is found at the INM, where it affects the formation of membrane bridges with nLDs and the architecture of the periplasmic space during nLD production.

### INM Lipid Composition Is Remodeled during NE Growth

**Figure S7:**
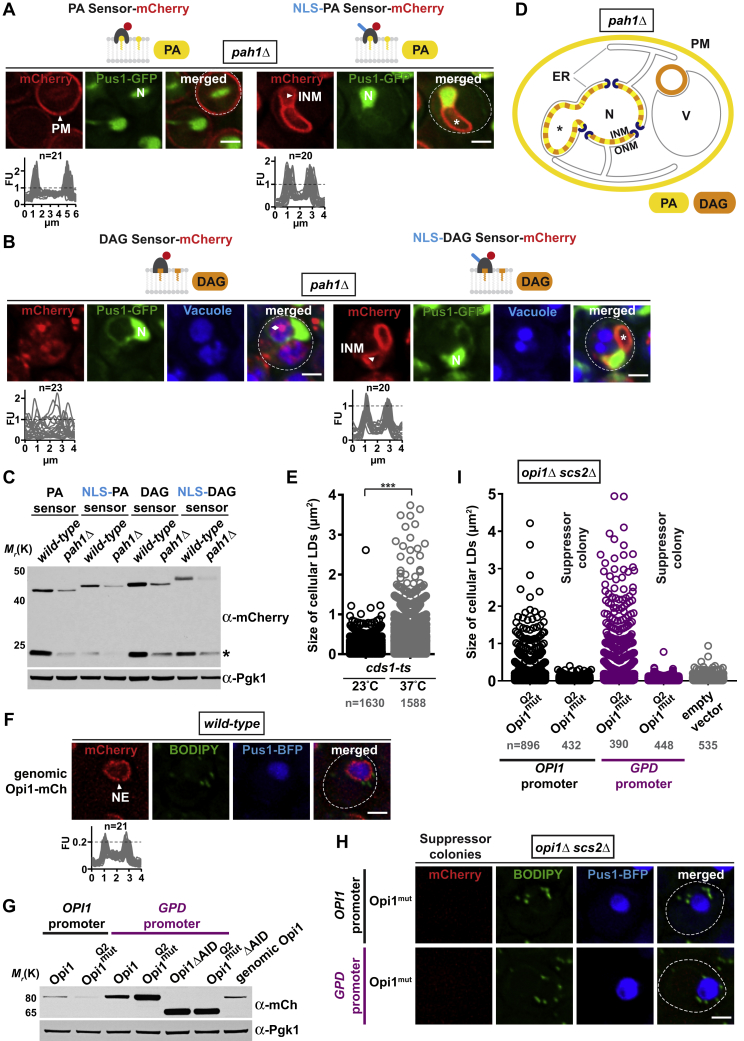
NE Growth Is Linked to an Increase of Phosphatidic Acid at the INM, Related to Figure 1, 6, 7, and Discussion (A) Live imaging of *pah1Δ* cells expressing the indicated plasmid-based PA-mCherry sensors and the genomically integrated nucleoplasmic marker Pus1-GFP. *PAH1* deletion induces nuclear membrane proliferation and nuclear expansion, which can lead to the engulfment of cytoplasmic material (marked by asterisk) as shown by the absence of Pus1 staining and earlier studies (Santos-Rosa et al., 2005). Line-scans were generated across the whole cell (left) or the nucleus (right). n indicates the number of randomly selected cells, y axis: Arbitrary Fluorescence Units, FU; x axis: distance in μm. For comparison the FU value 1 is marked with a horizontal dashed line. Plasma membrane, PM; nucleus, N; inner nuclear membrane, INM. Scale bar: 2 μm. (B) Live imaging of the indicated DAG sensors in *pah1Δ* cells. DAG sensor-reactive material (diamond), which often overlapped with the vacuole and may represent aberrant membrane structures, is frequently seen in *pah1Δ* cells. Vacuoles are stained with CellTracker Blue. Line-scans were generated across the vacuole (left) or the nucleus (right). Inner nuclear membrane, INM; nucleus, N. Asterisk indicates NE expansion. Scale bar: 2 μm. (C) Sensor protein levels were analyzed in wild-type and *pah1Δ* cells. Denaturing extracts were prepared and immunoblotted with an anti-mCherry antibody directed against the sensors and with an anti-Pgk1 antibody as a loading control. Sensor expression was reduced in the *pah1Δ* mutant. Asterisk indicates degradation product. (D) Schematic localization of major PA and DAG pools in *pah1Δ* cells as detected by lipid biosensors. Asterisk marks NE expansion. DAG-positive structures of unknown origin overlap with vacuoles. Inner nuclear membrane, INM; outer nuclear membrane, ONM; nucleus, N; endoplasmic reticulum, ER; plasma membrane, PM; vacuole, V. (E) Automated quantification of lipid droplet size in *cds1-ts* cells grown at 23°C or 37°C for 4 hours. After setting identical fluorescence intensity thresholds, circular BODIPY structures were automatically selected and quantified in ImageJ. Number of analyzed cells is indicated. p value (^∗∗∗^ < 0.001) was determined by Wilcoxon signed-rank test. (F) Live imaging of wild-type cells expressing genomically integrated Opi1-mCherry and plasmid-based Pus1-BFP. Lipid droplets are stained with BODIPY. The subcellular distribution of genomically expressed Opi1 is indistinguishable from plasmid-based Opi1. Opi1 interacts with the ER-protein Scs2 and labels mostly the NE. Line-scans quantify the fluorescent intensity across the nucleus. n indicates the number of randomly selected cells, y axis: Arbitrary Fluorescence Units, FU; x axis: distance in μm. The FU value 0.2 is marked with a horizontal dashed line. Nuclear envelope, NE. Scale bar: 2 μm. (G) Protein levels of the indicated plasmid-based Opi1-mCherry constructs expressed from different promoters in *opi1Δ scs2Δ* cells. Opi1 is expressed at similar levels from its genomic locus or from a plasmid harboring the endogenous *OPI1* promoter. Denaturing extracts were prepared and immunoblotted with an anti-mCherry antibody for Opi1 and with an anti-Pgk1 antibody as a loading control. (H) Characterization of fast-growing genetic suppressors, which emerged in the *opi1 Q2* mutant when expressed from the endogenous *OPI1* promoter (lane 2, -Ino, Figure 7D) or the stronger *GPD* promoter (lane 4, -Ino, Figure 7D). Individual colonies were picked from the plate and analyzed by fluorescent microscopy. Suppression of the growth defect likely stems from acquired mutations that abolish Opi1-mCherry expression as shown by the lack of mCherry fluorescence. Scale bar: 2 μm. (I) Automated quantification of lipid droplet size in (H) shows that suppression of the Opi1 Q2 mutant growth defect correlates with a reduction of cellular LD size. Suppressor colonies were compared to the respective Opi1 Q2 mutants shown in Figure 7B and an empty vector control. After setting identical fluorescence intensity thresholds for all experiments, circular BODIPY structures were automatically selected and quantified in ImageJ. Number of analyzed cells is indicated.

The finding of lipid storage initiating from the INM is striking. Since PA can be channeled into either lipid storage or lipid biosynthesis (Figure 1A), we asked whether INM lipids also change during membrane growth. We deleted Pah1, which increases cellular PA and induces excessive NE proliferation likely because PA is redirected toward phospholipid synthesis (Figure 1A) (Barbosa et al., 2015, Han et al., 2006, Santos-Rosa et al., 2005). In contrast to wild-type cells, the NLS-bearing PA sensor relocalized from the nuclear interior to the INM in *pah1Δ* cells, whereas the PA sensor without exogenous NLS remained at the plasma membrane and did not significantly label the ONM and ER (Figure S7A; protein levels in Figure S7C). This suggests that the INM can be altered in its PA content in response to changes in membrane growth. The typical DAG staining of the vacuolar membrane was decreased in *pah1Δ* cells (Figure S7B), consistent with reduced Pah1-dependent DAG production (Han et al., 2006). Using the NLS-bearing DAG sensor, we could still detect DAG at the INM in *pah1Δ* cells (Figure S7B), consistent with lipidomic studies, which suggested residual DAG synthesis by other enzymatic pathways in *pah1Δ* cells (Chae et al., 2012). In sum, we demonstrate an increase of PA at the INM in a situation where membrane proliferation is stimulated (Figure S7D). Together with the localization of key lipid enzymes at the INM (Figures 3A and 3B), these findings support the notion that the INM is a metabolically active lipid territory, which is remodeled in its PA content during both membrane growth and lipid storage.

### Nuclear and Cytoplasmic Lipid Droplet Production Is Co-regulated by Opi1

**Figure 6:**
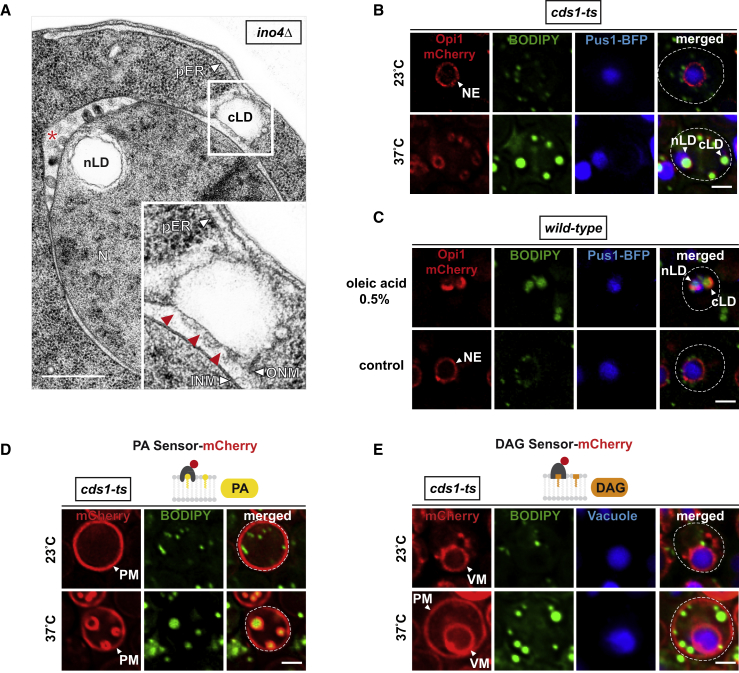
Coordinated Production and Size Control of Nuclear and Cytoplasmic Lipid Droplets (A) TEM analysis of an *ino4Δ* cell shows an nLD and a cLD. Inset shows magnified view of the boxed area with cLD connections to both ONM and pER. Multiple membrane contacts (red arrowheads) between the cLD and ONM are apparent. INM evaginations are labeled with red asterisk. Scale bar, 0.5 μm. (B) Live imaging of *cds1-ts* cells expressing Opi1-mCherry and the nucleoplasmic marker Pus1-BFP. Lipid droplets are stained with BODIPY. Cells were grown for 4 hr at the indicated temperatures. Scale bar, 2 μm. (C) Live imaging of Opi1-mCherry expressed in *opi1Δ* cells. Lipid droplets were stained with BODIPY. Cells were grown in oleic-acid-containing or control media. Scale bar, 2 μm. (D) Live imaging of *cds1-ts* cells expressing the PA-mCherry sensor. Lipid droplets were stained with BODIPY. Cells were grown for 4 hr at the indicated temperatures. Scale bar, 2 μm. (E) Live imaging of *cds1-ts* cells expressing the DAG-mCherry sensor. Lipid droplets were stained with BODIPY, vacuoles with CellTracker Blue. Cells were grown for 4 hr at the indicated temperatures. Note that upon Cds1 inhibition the sensor also uncovered a DAG pool at the plasma membrane (PM). VM, vacuolar membrane. Scale bar, 2 μm. See also Figure S7.

**Figure 7:**
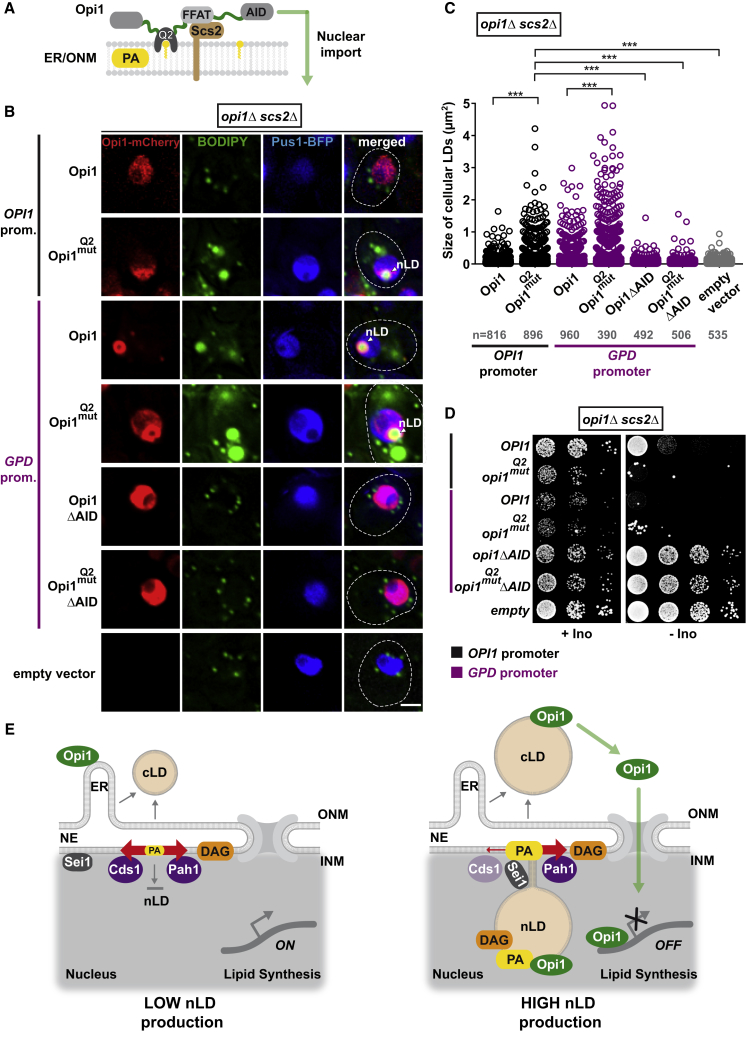
The Transcription Factor Opi1 Regulates Nuclear and Cytoplasmic Lipid Droplet Production (A) Cartoon of Opi1 domain organization and tethering to the endoplasmic reticulum (ER) and outer nuclear membrane (ONM) via its PA-sensing Q2 domain and the transmembrane protein Scs2. Opi1 contains an endogenous NLS, which partially overlaps with the Q2 domain (not depicted). FFAT, two phenylalanines (F) in an acidic tract motif; AID, activator interaction domain. (B) Live imaging of *opi1Δ scs2Δ* cells expressing the indicated plasmid-based Opi1-mCherry constructs or an empty vector. Lipid droplets were stained with BODIPY, the nucleoplasm with Pus1-BFP. Opi1 was either expressed from its endogenous promoter or overexpressed from the heterologous *GPD (TDH3)* promoter. Q2^mut^ indicates a mutation in the PA-binding domain, which reduces but does not abolish PA binding. For expression levels, see Figure S7G. Scale bar, 2 μm. (C) Automated quantification of lipid droplet size in (B). n = number of analyzed cells. ^∗∗∗^p value <0.001 was determined by ANOVA with post hoc Tukey HSD. (D) Growth analysis of *opi1Δ scs2Δ* cells expressing different plasmid-based constructs of Opi1-mCherry as in (B). Growth was followed on SDC-Ura-His and SDC-Ura-His-Inositol (-Ino) plates for 2 days at 30°C. Note that several fast-growing suppressor colonies emerged in the *opi1 Q2* mutant when expressed from the endogenous *OPI1* promoter (lane 2, -Ino) or the stronger *GPD* promoter (lane 4, -Ino). These genetic suppressors rescue the growth defect by abolishing Opi1 expression; see Figures S7H and S7I. (E) Model of nLD synthesis. At times of low nLD synthesis, cLD formation may predominate. This situation is favored by low PA levels at the INM due to turnover into DAG by Pah1 and/or CDP-DAG (not depicted) by Cds1. Expression of enzymes involved in cellular lipid biosynthesis is high due to the tethering of Opi to the ER. In contrast, nLD formation is stimulated by Opi1 translocation into the nucleus and repression of target genes (lipid synthesis OFF), a state that also induces cLD production. PA levels at the INM are increased, and PA and DAG become enriched on INM-tethered nLDs. Cds1 inactivation is a putative switch to channel PA into the storage branch of lipid metabolism. Opi1 partitioning on cLDs and nLDs constitutes a negative feedback mechanism for lipid droplet production. See also Figure S7.

The metabolic switch between membrane proliferation and lipid storage is controlled by Opi1, the master regulator of yeast phospholipid metabolism (Carman and Han, 2011, Loewen et al., 2004). The production of large nLDs in *cds1-ts* and *ino4Δ* cells coincided with the production of similarly sized cLDs (Figures 2E and 4D). cLDs are produced from the ONM and peripheral ER (Choudhary et al., 2015, Jacquier et al., 2011, Wilfling et al., 2013) (Figure 6A), and we show that nLDs are directly produced from the INM. These findings suggest a coordinated mechanism for lipid droplet synthesis and size control in the nuclear and cytoplasmic compartment. Opi1 is an ER-associated transcription factor that senses changes in cellular PA concentration, enabling feedback regulation of lipid metabolism. Opi1 binds PA via its Q2 domain (i.e., the domain that we used as a lipid biosensor) and at least one additional binding site. Opi1 also interacts with the ER protein Scs2 via its two phenylalanines (F) in an acidic tract motif (FFAT) domain (Figure 7A) (Loewen et al., 2004). Upon PA reduction, Opi1 translocates from the ER into the nucleus, where it represses the Ino2/4 transcriptional activator complex and hence induces expression of phospholipid metabolic genes such as *CDS1* (Figure 4A) (Carman and Han, 2011). Thus, changes in PA concentration are predicted to affect the location and repressor activity of Opi1. To address whether and how Opi1 might influence or be influenced by cytoplasmic and nuclear LD production, we first determined the localization of mCherry-tagged full-length Opi1 in *cds1-ts* cells grown at permissive (23°C) or restrictive (37°C) temperature (Figures 6B, S7E, and S7F). Opi1 localized mostly to the NE at 23°C, consistent with previous data (Loewen et al., 2004). Strikingly, Opi1 relocalized to the surface of both cLDs and nLDs upon *CDS1* inactivation (Figure 6B), and in wild-type cells grown with oleic acid (Figure 6C). The binding of Opi1 to nLDs and cLDs reflects their high PA content, as visualized by the PA sensors with and without an NLS (Figures 2B and 6D). Unlike PA, the DAG content seems higher on nLDs than cLDs under the conditions tested (Figures 3D and 6E).

These findings raised the question whether PA-mediated sequestration of Opi1 on lipid droplets influences Opi1 transcriptional activity. To test this, we altered Opi1 levels and localization and measured the effect on cellular lipid droplet size. We partially untethered Opi1 from the ER by deleting *SCS2*, such that Opi1 localization mainly depends on its ability to bind PA via its Q2 domain (Figure 7A). Accordingly, Opi1 expressed from its endogenous promoter now localized mainly to the nucleoplasm due to an endogenous NLS (Figure 7B). Interestingly, a mutant in the Opi1 Q2 domain (Q2^mut^), which decreases affinity toward PA, induced nLD production and caused an increase in cellular lipid droplet size (Figures 7B and 7C). We attribute this effect to increased repressor activity of Opi1 at Ino4/Ino2-occupied promoters, since the mutant Opi1 is no longer sequestered by PA-containing membranes such as the lipid droplet surface. We predicted that lipid droplet size should grow even further when the intranuclear dosage of Opi1 is increased. To this end, we expressed Opi1 variants from a strong heterologous promoter (*GPD*) in *scs2Δ* cells (Figure 7B; protein levels in Figure S7G). Notably, these cells exhibited large lipid droplets and the nLDs were coated by Opi1. We then assessed the Opi1 Q2 mutant expressed from the *GPD* promoter. This mutant produced even larger lipid droplets (Figure 7C) and Opi1 became more diffuse in the nucleoplasm (Figure 7B), likely because PA on the nLD surface is now poorly recognized. Ablating the Opi1 AID domain, which is required for its repressor activity, abolished nLD production in *scs2Δ* cells and lowered global lipid droplet size to a comparable level as the deletion of *OPI1* (Figures 7B and 7C). As a further functional readout, we measured growth of the Opi1 mutants on media, which lack inositol. Inositol auxotrophy is an indicator of Opi1 function (Loewen et al., 2004). Indeed, inositol auxotrophy was most pronounced in cells with high Opi1 repressor activity (Figure 7D) in line with the effects on lipid droplet size. Taken together, we show that Opi1 regulates both nLD and cLD formation. We propose that Opi1 absorption onto PA-rich nLDs and cLDs may reduce its intranuclear repressive activity, providing feedback control on lipid storage at the INM.

## Discussion

We have discovered active lipid metabolism at the INM and demonstrate a capacity of the INM for lipid storage via nuclear lipid droplet formation. This opens avenues for investigating the function of nuclear energy storage, as well as links between INM lipid metabolism and gene regulation.

### Metabolic Activity of the INM

The entry point for the identification of a distinct lipid metabolism of the INM was our finding that a DAG-specific sensor bound to the INM, but not the ONM/ER (Figure 1D). We cannot formally rule out that the sensor’s affinity is influenced by the nucleoplasmic or cytoplasmic environment. However, we were unable to find evidence that the DAG sensor could not bind the ONM/ER due to its limiting concentration (Figure S2D), or that DAG sensor binding to the INM is due to exclusion from chromatin or unspecific interactions with INM proteins (Figure S2C). Hence, a DAG asymmetry across the NE is a likely scenario. We hypothesized that the differential recognition of DAG across the NE may reflect a physiologically relevant lipid metabolic activity at the INM. Indeed, we found that the INM can be tuned for lipid storage by environmental and genetic perturbations (Figures 2G and 4B). An emerging question now is how cells establish an asymmetric lipid distribution across the NE despite the continuity of the ONM with the INM. Based on our finding of multiple lipid metabolizing enzymes at the INM, we propose that different metabolic activities between the ER/ONM and INM can create distinct lipid compositions. Hence, asymmetry may arise because the INM is topologically insulated from the ER/ONM by NPCs, which regulate which enzymes reach the INM. Moreover, all lipid traffic between ONM and INM must pass through the pore membrane beneath NPCs. NPCs may influence lipid traffic by hosting special enzymes or other “lipid gating” mechanisms that avoid mixing of the INM and ONM lipid environments.

### Function of Specific INM Lipids

Our study raises questions about the potential functions of PA and DAG at the INM. Why would the INM of exponentially growing yeast cells exhibit a low PA to DAG ratio? We suggest that PA is kept low at the INM by high turnover, which is mediated by the activities of Pah1 and Cds1 (Figure 7E). High PA levels at the INM either correlate with strong NE proliferation or with strong nLD formation, depending on which branch of lipid metabolism is active. Thus, a low PA level may reflect a dynamic steady state that can be tuned toward proliferation or storage.

Does the apparent high abundance of DAG at the INM only reflect the turnover of PA? Probably not; DAG has special physicochemical properties, which could play multiple physiologically important roles at the INM. DAG is a conically shaped lipid (Thiam et al., 2013), and this creates lipid packing defects in bilayers, which could favor the binding of specific INM proteins. DAG also facilitates the fusion of lipid bilayers, for example, during vesicle budding (Fernández-Ulibarri et al., 2007). Interphase NPC biogenesis requires a fusion between the ONM and INM, which is not understood (Ungricht and Kutay, 2017). It will be interesting to examine whether DAG at the INM influences membrane remodeling and NPC assembly. Moreover, DAG becomes enriched on nLDs, suggesting that the INM reservoir of DAG could play a role in lipid droplet biogenesis in conjunction with PA. The conically shaped DAG may not only act as a surfactant, but also as fusogenic lipid during lipid droplet coalescence (Thiam et al., 2013) and as an inducer of negative curvature, which is present at nLD-INM membrane bridges. Finally, DAG is an important second messenger in cell signaling. The DAG-dependent kinase PKCβ, from which our DAG sensor is derived, phosphorylates histone H3 to regulate gene expression (Metzger et al., 2010). Whether DAG-dependent PKCβ signaling occurs inside the nucleus remains to be determined. Given that PA has similar effects on membrane structure to DAG (Thiam et al., 2013), investigating DAG and PA chemistry at the INM can illuminate new biological roles of these versatile lipid precursors and their potential roles in nuclear lipid signaling.

### Metabolic Adaptability of the INM

We identified enzymes of both branches of lipid metabolism at the INM. With regards to membrane proliferation, this could imply that NE growth during the cell cycle relies on local lipid synthesis and turnover at the INM in addition to lipid delivery from the ER/ONM. The physiologic importance of lipid storage at the INM is underscored by the fact that a surplus of fatty acids induces nLD formation (Figure 2G). nLD production appears to be triggered by mechanisms that increase PA levels at the INM and draw PA into the storage branch (Figure 7E). nLD and cLD sizes seem to be co-regulated and are governed by the amount of Opi1 inside the nucleus, where Opi1 represses the promoters of genes involved in phospholipid synthesis (Figure 7E). Hypothetically, Opi1 could turn on lipid droplet production by inhibiting *CDS1* expression, thereby shunting PA into TAG synthesis. Why then is Opi1 sequestered on both cLDs and nLDs via its PA-sensing domain? This association seems to restrict access of Opi1 to target genes and thereby restrain Opi1’s ability to induce lipid droplet formation. We propose that nLDs act as signaling platforms for transcription factors in spatial proximity to target promoters. By forming a classical negative feedback loop, this could buffer short-term fluctuations of nutrients and promote lipid homeostasis.

### Function and Dysfunction of INM Lipid Storage

What is the role of nLDs beyond their involvement in Opi1-dependent gene expression? The presence of nLD-INM bridges strongly suggests that nLDs communicate with the INM. Besides receiving lipids, nLDs may return lipids to the INM in times of increased demand, for example, during resumption of the cell cycle after stationary phase. cLDs were implicated in the neutralization of cytotoxic free fatty acids as well as the storage of misfolded hydrophobic proteins and histones (Welte, 2015). It will be interesting to examine, whether nLDs act as a storage site for nuclear proteins and, more generally, as a signaling platform for proteins with phospholipid-binding domains (Lemmon, 2008). Based on our work, lipid droplet function can now be comprehensively addressed in the context of nuclear biology taking advantage of the experimental versatility of *S. cerevisiae*. We have shown that yeast Seipin is required for the formation of proper membrane bridges between the INM and nLDs. Of note, human Seipin is mutated in Berardinelli-Seip congenital lipodystrophy type 2, which is characterized by a severe loss of body fat, ectopic fat deposition, and a deranged overall metabolism (Szymanski et al., 2007). Additionally, mutations in Lipin, the human ortholog of yeast Pah1, cause lipodystrophy in mice (Péterfy et al., 2001). The presence of Sei1 and Pah1 at the yeast INM raises the question whether their INM function is conserved between yeast and humans and, if so, which role the INM plays in the pathogenesis of human metabolic diseases (Krahmer et al., 2013).

In sum, we suggest that the INM is not merely a remote province of the ER, spatially isolated and dependent on lipid supplies from the ER. Instead, we identify the INM as a territory with its own lipid metabolism and striking metabolic adaptability. This conceptual framework is testable in more diverse genetic perturbations, additional biological settings, and across evolution.

## STAR★Methods

### Key Resources Table

**Table:** 

REAGENT or RESOURCE	SOURCE	IDENTIFIER
**Antibodies**		

Mouse monoclonal anti-mCherry	Abcam	Cat.#ab125096
Mouse monoclonal anti-GFP (clones 7.1 and 13.1)	Roche	Cat.#ab11814460001
Mouse monoclonal anti-Pgk1	Abcam	Cat.#ab113687
Peroxidase AffiniPure Goat anti-Mouse IgG	Jackson ImmunoResearch	Cat.#115035008
Rabbit polyclonal anti-GFP	Abcam	Cat.#ab6556
Goat anti-Rabbit IgG coupled with 6 nm gold	Aurion	Cat.#806.011

**Chemicals, Peptides, and Recombinant Proteins**		

BODIPY 493/503	Thermo Fisher Scientific	Cat.#D3922
CellTracker Blue CMAC Dye	Thermo Fisher Scientific	Cat.#C2110
TWEEN 80	Sigma-Aldrich	Cat.#P1754
Oleic acid	Sigma-Aldrich	Cat.#O1008
Rapamycin	LC Laboratories	Cat.#R-5000
Concanavalin A	Sigma-Aldrich	Cat.#C2010

**Experimental Models: Organisms/Strains**		

*S. cerevisiae* strain BY4741, genotype: *MATa; ura3*Δ*0; leu2*Δ*0; his3*Δ*1; met15*Δ*0*	Euroscarf	Y00000
*S. cerevisiae* strain Nup188-GFP, genotype: *MATa; ura3*Δ*0; leu2Δ0; his3*Δ*1; met15*Δ*0; NUP188-GFP::HIS3MX6*	(Mészáros et al., 2015)	N/A
*S. cerevisiae* strain *cds1-ts*, genotype: *MATa; ura3*Δ*0; leu2*Δ*0; his3*Δ*1; met15*Δ*0; cds1-ts::KanR*	(Li et al., 2011)	N/A
*S. cerevisiae* strain *pah1*Δ, genotype: *MATa; ura3*Δ*0; leu2*Δ*0; his3*Δ*1; met15*Δ*0; pah1Δ::natNT2*	This paper	N/A
*S. cerevisiae* strain *pah1*Δ Pus1-GFP, genotype: *MATa; ura3*Δ*0; leu2*Δ*0; his3*Δ*1; met15*Δ*0; pah1Δ::natNT2; PUS1-GFP::kanMX4*	This paper	N/A
*S. cerevisiae* strain *kap123*Δ Nup188-GFP, genotype: *MATa; ura3*Δ*0; leu2*Δ*0; his3*Δ*1; met15*Δ*0; kap123Δ::kanMX4; NUP188-GFP::natNT2*	(Mészáros et al., 2015)	N/A
*S. cerevisiae* strain *cds1-ts* Nup188-GFP, genotype: *MATa; ura3*Δ*0; leu2*Δ*0; his3*Δ*1; met15*Δ*0; cds1-ts::KanR; NUP188-GFP::natNT2*	This paper	N/A
*S. cerevisiae* strain *cds1-ts* Tgl1-GFP, genotype: *MATa; ura3*Δ*0; leu2*Δ*0; his3*Δ*1; met15*Δ*0; cds1-ts::KanR; TGL1-GFP::natNT2*	This paper	N/A
*S. cerevisiae* strain *cds1-ts* Tgl4-GFP, genotype: *MATa; ura3*Δ*0; leu2*Δ*0; his3*Δ*1; met15*Δ*0; cds1-ts::KanR; TGL4-GFP::natNT2*	This paper	N/A
*S. cerevisiae* strain *cds1-ts* Tgl5-GFP, genotype: *MATa; ura3*Δ*0; leu2*Δ*0; his3*Δ*1; met15*Δ*0; cds1-ts::KanR; TGL5-GFP::natNT2*	This paper	N/A
*S. cerevisiae* strain *cds1-ts* Yju3-GFP, genotype: *MATa; ura3*Δ*0; leu2*Δ*0; his3*Δ*1; met15*Δ*0; cds1-ts::KanR; YJU3-GFP::natNT2*	This paper	N/A
*S. cerevisiae* strain Opi1-mCh, genotype: *MATa; ura3*Δ*0; leu2*Δ*0; his3*Δ*1; met15*Δ*0; OPI1-mCh::natNT2*	This paper	N/A
*S. cerevisiae* strain *dgk1*Δ, genotype: *MATa; ura3*Δ*0; leu2*Δ*0; his3*Δ*1; met15*Δ*0; dgk1Δ::kanMX4*	Euroscarf	Y01608
*S. cerevisiae* strain *ino2*Δ, genotype: *MATa; ura3*Δ*0; leu2*Δ*0; his3*Δ*1; met15*Δ*0; ino2Δ::kanMX4*	Euroscarf	Y04057
*S. cerevisiae* strain *ino4*Δ, genotype: *MATa; ura3*Δ*0; leu2*Δ*0; his3*Δ*1; met15*Δ*0; ino4Δ::kanMX4*	Euroscarf	Y06258
*S. cerevisiae* strain *ino4*Δ *sei1*Δ, genotype: *MATa; ura3*Δ*0; leu2*Δ*0; his3*Δ*1; met15*Δ*0; ino4Δ::kanMX4; sei1Δ::natNT2*	This paper	N/A
*S. cerevisiae* strain *opi1*Δ, genotype: *MATa; ura3*Δ*0; leu2*Δ*0; his3*Δ*1; met15*Δ*0; opi1Δ::kanMX4*	Euroscarf	Y00943
*S. cerevisiae* strain *opi1*Δ *scs2*Δ, genotype: *MATa; ura3*Δ*0; leu2*Δ*0; his3*Δ*1; met15*Δ*0; opi1Δ::kanMX4; scs2Δ::natNT2*	This paper	N/A
*S. cerevisiae* strain Pma1-2xFKBP12, *Matα; leu2-3,112; trp1-1; can1-100; ura3-1; ade2-1; his3-11,15; tor1-1; fpr1::NAT; PMA1-2 × FKBP12::TRP1*	Euroscarf	Y40342

**Recombinant DNA**		

Plasmid: Opi1 Q2-mCh: *pRS316-CYC1prom-OPI1 Q2-mCh*	This paper	N/A
Plasmid: *GPD prom*-Opi1 Q2-mCh: *pRS316-GPDprom-OPI1 Q2-mCh*	This paper	N/A
Plasmid: Opi1 Q2^mut^-mCh: *pRS316-CYC1prom-opi1 Q2 (L124R, Y127A, L129R, M131A, I133R, K136A, K137A, R138A)-mCh*	This paper	N/A
Plasmid: NLS-Opi1 Q2-mCh: *pRS316-CYC1prom-NUP60(1-24)-OPI1 Q2-mCh*	This paper	N/A
Plasmid: NLS-Opi1 Q2^mut^-mCh: *pRS316-CYC1prom-NUP60(1-24)-opi1 Q2(L124R, Y127A, L129R, M131A, I133R, K136A, K137A, R138A)-mCh*	This paper	N/A
Plasmid: NLS-Opi1 Q2-mGFP: *pRS316-CYC1prom-NUP60(1-24)-OPI1 Q2-mGFP*	This paper	N/A
Plasmid: NLS-Opi1 Q2-mCh: *pRS315-CYC1prom-NUP60(1-24)-OPI1 Q2-mCh*	This paper	N/A
Plasmid: SV40 NLS-Opi1 Q2-mCh: *pRS316-CYC1prom-SV40 NLS-OPI1 Q2-mCh*	This paper	N/A
Plasmid: C1a+C1b-mCh: *pRS316-CYC1prom-C1a+C1b-mCh*	This paper	N/A
Plasmid: *GPDprom*-C1a+C1b-mCh: *pRS316-GPDprom-C1a+C1b-mCh*	This paper	N/A
Plasmid: *GAL1prom*-C1a+C1b-mCh: *pRS316-GAL1prom-C1a+C1b-mCh*	This paper	N/A
Plasmid: C1a+C1b^mut^-mCh: *pRS316-CYC1prom-C1a+C1b(Q63E, Q128E)-mCh*	This paper	N/A
Plasmid: NLS-C1a+C1b-mCh: *pRS316-CYC1prom-NUP60(1-24)-C1a+C1b-mCh*	This paper	N/A
Plasmid: NLS-C1a+C1b^mut^-mCh: *pRS316-CYC1prom-NUP60(1-24)- C1a+C1b(Q63E, Q128E)-mCh*	This paper	N/A
Plasmid: NLS-C1a+C1b-mGFP: *pRS316-CYC1prom-NUP60(1-24)-C1a+C1b-mGFP*	This paper	N/A
Plasmid: SV40 NLS-C1a+C1b-mCh: *pRS316-CYC1prom-SV40 NLS-C1a+C1b-mCh*	This paper	N/A
Plasmid: Spo20-mCh: *pRS316-CYC1prom-SPO20(51-91)-mCh*	This paper	N/A
Plasmid: Spo20^mut^-mCh: *pRS316-CYC1prom-spo20(51-91)K66E, K68E, R71E, K73E-mCh*	This paper	N/A
Plasmid: NLS-Spo20-mCh: *pRS316-CYC1prom-NUP60(1-24)-SPO20(51-91)-mCh*	This paper	N/A
Plasmid: NLS-Spo20^mut^-mCh: *pRS316-CYC1prom-NUP60(1-24)-spo20(51-91)K66E, K68E, R71E, K73E-mCh*	This paper	N/A
Plasmid: Nup60-VN: *pRS315-NUP60prom-NUP60-GS-VN*	This paper	N/A
Plasmid: Pus1-VN: *pRS315-PUS1prom-PUS1-GS-VN*	This paper	N/A
Plasmid: Opi1 Q2-VC: *pRS313-CYC1prom-OPI1 Q2-5xGS-VC*	This paper	N/A
Plasmid: NLS-Opi1 Q2-VC: *pRS313-CYC1prom-NUP60(1-24)-OPI1-Q2-5xGS-VC*	This paper	N/A
Plasmid: C1a+C1b-VC: *pRS313-CYC1prom-C1a+C1b-5xGS-VC*	This paper	N/A
Plasmid: NLS-C1a+C1b-VC: *pRS313-CYC1prom-NUP60(1-24)-C1a+C1b-5xGS-VC*	This paper	N/A
Plasmid: VC-Cds1: *pRS313-CDS1prom-VC-GS-CDS1*	This paper	N/A
Plasmid: VC-Dgk1: *pRS313-ADH1prom-VC-GS-DGK1*	This paper	N/A
Plasmid: Pah1-VC: *pRS313-GPDprom-PAH1-5xGS-VC*	This paper	N/A
Plasmid: NES-Pah1-VC: *pRS313-GPDprom-RNA1(316-357)-PAH1-5xGS-VC*	This paper	N/A
Plasmid: mNES-Pah1-VC: *pRS313-GPDprom-RNA1(316-357)L320A, L323A, L326A, I328A, L340A, L342A-PAH1-5xGS-VC*	This paper	N/A
Plasmid: Sei1-VC: *pRS313-ADH1prom-SEI1-5xGS-VC*	This paper	N/A
Plasmid: *OPI1prom*-Opi1-mCh: *pRS316-OPI1prom-OPI1-mCh*	This paper	N/A
Plasmid*: OPI1p*rom-Opi1^mut^-mCh: *pRS316-OPI1prom-opi1(L124R, Y127A, L129R, M131A, I133R, K136A, K137A, R138A)-mCh*	This paper	N/A
Plasmid: *GPDprom*-Opi1-mCh: *pRS316-GPDprom-OPI1-mCh*	This paper	N/A
Plasmid: *GPDprom*-Opi1^mut^-mCh: *pRS316-GPDprom-opi1(L124R, Y127A, L129R, M131A, I133R, K136A, K137A, R138A)-mCh*	This paper	N/A
Plasmid: *GPDprom*-Opi1ΔAID-mCh: *pRS316-GPDprom-OPI1(1-273)-mCh*	This paper	N/A
Plasmid: *GPDprom*-Opi1^mut^ΔAID-mCh: *pRS316-GPDprom-opi1(1-273)(L124R, Y127A, L129R, M131A, I133R, K136A, K137A, R138A)-mCh*	This paper	N/A
Plasmid: Pus1-BFP: *pRS313-PUS1prom-PUS1-BFP*	This paper	N/A
Plasmid: mGFP-Dgk1: *pRS313-GAL1prom-mGFP-DGK1*	This paper	N/A
Plasmid: mGFP-NLS-Dgk1: *pRS313-GAL1prom-mGFP-HEH2(93-317)-DGK1*	This paper	N/A
Plasmid: mGFP-NLS-Dgk1 D177A: *pRS313-GAL1prom-mGFP-HEH2(93-317)-dgk1 D177A*	This paper	N/A
Plasmid: mGFP-Cds1: *pRS313-GPDprom-mGFP-CDS1*	This paper	N/A
Plasmid: Pah1-mGFP: *pRS313-GPDprom-PAH1-mGFP*	This paper	N/A
Plasmid: Pah1-FRB-GFP: *pRS313-GPDprom-PAH1-FRB-GFP*	This paper	N/A
Plasmid: Pah1 7A-FRB-GFP: *pRS313-GPDprom-pah1 S110A, S114A, S168A, S602A, T723A, S744A, S748A-FRB-GFP*	This paper	N/A

**Software and Algorithms**		

ImageJ	NIH	https://imagej.nih.gov/ij/
GraphPad Prism	GraphPad	https://www.graphpad.com/
IMOD	(Kremer et al., 1996)	http://bio3d.colorado.edu/imod/
softWoRX	GE Healthcare	N/A
RStudio	RStudio	https://www.rstudio.com/

### Contact for Reagent and Resource Sharing

Further information and requests for resources and reagents should be directed to and will be fulfilled by the Lead Contact, Alwin Köhler (alwin.koehler@mfpl.ac.at).

### Experimental Model and Subject Details

#### Strains and media

All yeast strains used in this study are listed in the Key Resources Table. Genes in yeast were tagged/deleted by a standard one-step PCR-based technique. Microbiological techniques followed standard procedures. Cells were grown in standard yeast extract peptone dextrose (YPD) or when transformed with plasmids in selective synthetic dextrose complete (SDC) drop-out media. Oleic acid-containing growth media were prepared from standard SDC media containing 0.1% glucose, 0.5% oleic acid, 1% Tween 80. Oleic acid was omitted in the negative control. To induce protein production from the *GAL1* promoter, cells were grown exponentially in media containing 2% raffinose before adding galactose to a final concentration of 2% and further growth for 4 hr (for Dgk1, Figures S3B–S3E) or 3.5 hr (for DAG sensor, Figure S2D). For rapamycin-dependent targeting of Pah1 to the plasma membrane, cells were grown in synthetic media supplemented with adenine (0.2 mg/mL). Microscopy was performed 1 hr after rapamycin (1 μg/mL) addition.

### Method Details

#### Sensor design and construction

For the Opi1 Q2 PA sensor, a fragment comprising Opi1 aa103-189 was amplified from yeast genomic DNA. This fragment corresponds to the reported PA binding domain and contains an endogenous NLS sequence (aa109-112; KRQK) (Loewen et al., 2004). To create a PA-binding deficient mutant of Opi1 Q2, the following mutations were introduced based on a previous study (Loewen et al., 2004): L124R, Y127A, L129R, M131A, I133R, K136A, K137A, R138A. For the PKCβ C1a+C1b DAG sensor, a fragment comprising aa31-158 was amplified from cDNA. DAG recognition is predicted to depend on specific residues (Lučić et al., 2016), which were simultaneously mutated to abolish DAG sensing (Q63E in the C1a domain and Q128E in the C1b domain). All sensors are constructed in a modular fashion allowing exchange of promoters and fluorophores, NLS-sequences corresponding to Nup60 aa1-24 were introduced by PCR.

#### Live-Cell Imaging of Yeast

Exponentially growing cells were immobilized on microscope slides with agarose pads and imaged on a DeltaVision Elite microscope (GE Healthcare) in a temperature-controlled chamber. Images were acquired with a 60x oil immersion objective and recorded with a CoolSNAP HQ2 CCD camera (Photometrics). Deconvolution was carried out using softWoRx software (GE Healthcare). Images were processed with ImageJ. Cell contours were marked with a dashed white line based on brightfield imaging. To stain lipid droplets or vacuoles, BODIPY 493/503 (final concentration 5.7 μM) or CellTracker Blue (final concentration 10 μM) was added, respectively, and cells were imaged after 20 min. For time-lapse imaging, yeast cells were immobilized with Concanavalin A (Sigma-Aldrich) on μ-Slide (8-Well Glass Bottom, Ibidi).

#### Yeast growth assay

Cells were grown exponentially, harvested and resuspended to a final OD_600_ of 0.5. 10-fold serial dilutions were prepared, spotted on appropriate plates and incubated at 23, 30 or 37°C.

#### Immunogold Electron Microscopy

Cells were fixed in 2% paraformaldehyde and 0.2% glutaraldehyde (both from EMS) in 0.1 M Sörensen phosphate buffer (pH 7.4) for 2 hr at room temperature (RT), then overnight at 4°C. The cell pellet was embedded in 12% gelatine and cut into 1 mm^3^ blocks, which were immersed in 2.3 M sucrose overnight at 4°C. These blocks were mounted onto a Leica specimen carrier (Leica Microsystems) and frozen in liquid nitrogen. Frozen blocks were cut into ultra-thin sections at a nominal thickness of 60 nm at −120°C with a Leica UCT/FCS cryo-ultramicrotome (Leica Microsystems). A mixture of 2% methylcellulose and 2.3 M sucrose (1:1 ratio) was used as a pick-up solution. Sections were picked up onto 200 mesh Ni grids (Gilder Grids) with a carbon coated formvar film (Tokuyasu method). For immunolabeling, grids were placed on plates with solidified 2% gelatine and warmed up to 37°C for 20 min to remove the pick-up solution. After quenching of free aldehyde-groups with glycin (0.1% for 15 min), a blocking step with 1% BSA (fraction V) in 0.1 M Sörensen phosphate buffer (pH 7.4) was performed for 30 min. The grids were incubated in primary antibody, rabbit anti-GFP (Abcam), diluted 1:250 in 0.1 M Sörensen phosphate buffer containing 0.1% BSA over night at 4°C. Subsequently, a goat anti-rabbit secondary antibody coupled with 6 nm gold (GAR 6 nm, Aurion), diluted 1:20 in 0.1 M Sörensen phosphate buffer containing 0.1% BSA, was applied for 2 hr at RT. The sections were stained with 4% uranyl acetate (Merck) and 2% methylcellulose in a ratio of 1:9 on ice. All labeling steps were performed in a wet chamber. The sections were inspected using a FEI Morgagni 268D TEM (FEI) operated at 80 kV. Electron micrographs were acquired using an 11 megapixel Morada CCD camera from Olympus-SIS.

#### Cryo-preparation and Transmission electron microscopy (TEM)

*ino4Δ* and *ino2Δ* cells were grown at 30°C, whereas the *cds1-ts* strain was grown at 37°C for 4 hr in synthetic media prior to TEM analysis. Pelleted cells were mixed 1:1 with 10% BSA, used as a filler, and transferred into the 100 μm cavity of a 3 mm aluminum specimen carrier. This carrier was sandwiched with a flat 3 mm aluminum carrier and immediately high pressure frozen in a HPF Compact 01 (both carriers and high pressure freezer from Engineering Office M. Wohlwend GmbH). The frozen samples were subsequently transferred into a Leica EM AFS-2 freeze substitution unit (Leica Microsystems). Over a period of 4 days, samples were substituted in a medium of acetone containing 2% osmium tetroxide (Agar Scientific), 0.2% uranyl acetate and 2%–5% water. Freeze substitution was performed according to the following protocol: 30-40 hr at –90°C, warm up at a rate of 2°C per hour to –54°C, 8 hr at –54°C, warm up at a rate of 5°C per hour to –24, 15 hr at –24°C, warm up at a rate of 5°C per hour to 0°C, 2 hr at 0°C. At 0°C samples were taken out and washed 3 times in anhydrous acetone (on ice) and infiltrated with Agar 100 Epoxy resin (Agar Scientific), in a graded series of acetone and resin over a period of 3 days. Polymerization took place at 60°C. Ultra-thin sections with a nominal thickness of 70 nm were cut using a Leica UCT ultramicrotome (Leica Microsystems) and post-stained with 2% aqueous uranyl acetate and Reynold’s lead citrate. Examination regions on the sections were randomly selected and inspected with a FEI Morgagni 268D (FEI) operated at 80 kV. Digital images were acquired using an 11 megapixel Morada CCD camera (Olympus-SIS).

#### Electron Tomography

For room temperature electron tomography, *ino4Δ* cells were prepared as described in the previous section, except that 150-250 nm sections were cut on a Leica UCT ultramicrotome (Leica Microsystems). After collecting the sections on a 50mesh Cu/Pd grid (Gilder Grids), previously coated with a supporting film of formvar, the section was covered on both sides with 10 nm gold (Aurion) by incubating the grid in a drop of concentrated gold solution for 3 min. Tilt series were acquired at a Tecnai G2 20 microscope (FEI) equipped with an Eagle 4k HS CCD camera (FEI) and operated at 200 kV. Dual axis tilt series were collected with a tilting range from −60° to +60° each at 1° increment. For data acquisition, processing and modeling the IMOD software (Kremer et al., 1996) from the Boulder Laboratory for 3D Electron Microscopy of Cells was used.

#### Immunoblotting

Yeast whole-cell lysates were prepared, normalized for protein concentration and analyzed by western blotting according to standard procedures. Antibodies were used according to the manufacturer’s instructions.

### Quantification and Statistical Analysis

To manually quantify the percentage of cells with nLDs, the following criteria were used for nLD classification: the NLS-PA sensor co-localizes with a BODIPY-stained structure and this structure exhibits a round shape (for Figure 2D at least 200 cells for each condition and for Figure 4C wild-type n = 150, *ino4Δ* n = 400). The LD diameter was then measured using ImageJ (Figures 2E and 4D).

To manually quantify the NLS-PA sensor localization in cells grown with oleic acid (Figure 2H), the following criteria were used: nLDs were classified according to the definition above. The sensor was classified as exhibiting an INM localization, whenever cells had no nLD, but exhibited a higher fluorescent intensity at the NE as compared to the nucleoplasm. The peak fluorescent intensity of the NE was measured by drawing a line across the NE using ImageJ. If the peak intensity at the NE was at least 1.5 times higher than the nucleoplasmic fluorescent intensity, the sensor localization was classified as “INM,” otherwise as “nucleoplasmic” (n = 195 cells treated with oleic acid and n = 140 control cells).

Automated quantification of cellular LD area was performed with ImageJ (Figures 7C, S4C, S7E, and S7I). The lower fluorescent intensity threshold was chosen such that BODIPY-stained LDs appeared as single, non-clustered circular particles. Identical thresholds were applied to all experimental conditions. Particles with a circularity index of 0.7-1.0, were used to compare the area of LDs of different yeast cells (at least 390 cells were analyzed for each condition).

To quantify sensor fluorescence intensity, a line was drawn across the organelle of interest and line-scan graphs were generated to measure the intensity value of the pixels (ImageJ software). Image acquisition parameters for images of the same experiment are always identical. Number of analyzed cells (n) is indicated in each figure.

Gold particle quantification was performed by counting particles within a 125 nm zone relative to the NE midline (INM and ONM side) or in the nucleoplasm (NP) (> 125 nm from NE midline). For Figure 3B, 381 gold particles (Pah1) and 602 particles (Cds1) from 8 nuclei were counted. For Figure S3D, 760 gold particles (Dgk1) and 877 particles (NLS-Dgk1) from 9 nuclei were counted.

Statistical significance was evaluated by Wilcoxon test or ANOVA analysis and Tukey HSD test using the GraphPad Prism and RStudio software. ^∗^ represents p < 0.05, ^∗∗^ p < 0.01, ^∗∗∗^ p < 0.001.
